# Dynamic single cell measurements of kinase activity by synthetic kinase activity relocation sensors

**DOI:** 10.1186/s12915-015-0163-z

**Published:** 2015-08-01

**Authors:** Eric Durandau, Delphine Aymoz, Serge Pelet

**Affiliations:** Department of Fundamental Microbiology, University of Lausanne, CH-1015 Lausanne, Switzerland

## Abstract

**Background:**

Mitogen activated protein kinases (MAPK) play an essential role in integrating extra-cellular signals and intra-cellular cues to allow cells to grow, adapt to stresses, or undergo apoptosis. Budding yeast serves as a powerful system to understand the fundamental regulatory mechanisms that allow these pathways to combine multiple signals and deliver an appropriate response. To fully comprehend the variability and dynamics of these signaling cascades, dynamic and quantitative single cell measurements are required. Microscopy is an ideal technique to obtain these data; however, novel assays have to be developed to measure the activity of these cascades.

**Results:**

We have generated fluorescent biosensors that allow the real-time measurement of kinase activity at the single cell level. Here, synthetic MAPK substrates were engineered to undergo nuclear-to-cytoplasmic relocation upon phosphorylation of a nuclear localization sequence. Combination of fluorescence microscopy and automated image analysis allows the quantification of the dynamics of kinase activity in hundreds of single cells. A large heterogeneity in the dynamics of MAPK activity between individual cells was measured. The variability in the mating pathway can be accounted for by differences in cell cycle stage, while, in the cell wall integrity pathway, the response to cell wall stress is independent of cell cycle stage.

**Conclusions:**

These synthetic kinase activity relocation sensors allow the quantification of kinase activity in live single cells. The modularity of the architecture of these reporters will allow their application in many other signaling cascades. These measurements will allow to uncover new dynamic behaviour that previously could not be observed in population level measurements.

**Electronic supplementary material:**

The online version of this article (doi:10.1186/s12915-015-0163-z) contains supplementary material, which is available to authorized users.

## Background

Signal transduction is an essential part of cellular life. Cells perceive changes in their environment and integrate these extra-cellular signals with intra-cellular cues to mount an appropriate response. This is achieved by signal transduction cascades, where receptors at the plasma membrane sense the surrounding medium and transmit this information to intracellular enzymes that modulate the activity of other proteins via post-translational modifications to induce a specific response.

The mitogen activated protein kinase (MAPK) pathways are a conserved family of signaling cascades which respond to a wide range of signals such as growth hormones, nutrient status, or stresses [1]. These pathways are activated by surface sensors (often G-protein coupled receptors), which transduce their information via membrane-associated proteins to the highest member of the MAPK cascade, the MAP kinase kinase kinase (MAP3K). In turn, the MAP3K phosphorylates the MAP kinase kinase (MAP2K), which finally doubly phosphorylates the MAPK to activate it. MAPKs have a large range of substrates both in the cytoplasm and in the nucleus, where they actively promote the transcription of new genes. Although these pathways are often viewed as linear signaling routes where a given output elicits a specific response, they are actually part of a complex signaling network where cross-activation and cross-inhibition mechanisms are readily found.

In the model organism *Saccharomyces cerevisiae*, four MAPK pathways are active in haploid cells: the mating pathway, the filamentous growth pathway, the high osmolarity glycerol (HOG) pathway, and the cell wall integrity (CWI) pathway (Additional file 1: Figure S1) [2]. The mating pathway is activated in haploid cells in response to pheromone (a- or α-factor). In MATa cells, the α-factor is sensed by the G-protein coupled receptor Ste2. The binding of the ligand results in the dissociation of the trimeric G-protein. Free Gβγ recruits the scaffold protein Ste5 to the plasma membrane, which promotes the activation of the MAP3K Ste11 and in turn activates the MAP2K Ste7. This kinase further activates two MAPKs, Fus3 and Kss1, both of which contribute to the mating response [3, 4].

While this pathway is a prototypical example of a growth response pathway, the CWI is more akin to a stress response cascade. Cell wall remodelling is constantly taking place during growth of the yeast. In addition, the CWI pathway is also acutely activated by environmental stresses such as heat, hypo-osmotic stress, or cell wall damaging agents [5]. The sensing mechanism of these stresses is not fully understood, it is nevertheless clear that they act via MAP3K Bck1, which activates the MAP2Ks Mkk1 and Mkk2, both of which phosphorylate the MAPK Mpk1 (Slt2). Mid2 and Wsc1 have been identified as the most important sensors for this pathway and transduce their status via Rho1 and Pkc1 to the MAPK cascade [5]; however, other stimuli take alternative routes. As an example, zymolyase (which consists of a cocktail of enzymes degrading the cell wall) activates the HOG pathway and, subsequently, its MAPK Hog1 activates Pkc1 via an unknown mechanism [6].

Despite its apparent simplicity, the budding yeast MAPK network shares multiple common features with its counterparts in higher eukaryotes, such as MAP3K or MAP2K, which can activate multiple downstream MAPKs depending on the stimulus. Extensive experimental evidence has demonstrated that these pathways are highly interdependent [7–9]. Moreover, these pathways are embedded within the global signaling network of the cells. Thus, they can regulate cell cycle progression, metabolism, or stress response, or in other instances be regulated by these same processes. The integration of these signals can lead to a variability in the response of individual cells to a given stimulus because the physiological state of each individual cells can vary [10–13]. Thus, dynamic and quantitative single cell measurements are required to untangle the complex regulatory network that controls the activity of MAPKs in a cell.

Therefore, we set out to design synthetic substrates for MAPKs that would relocate between the cytoplasm and the nucleus upon phosphorylation. These synthetic kinase activity relocation sensors (SKARS) were designed with a modular architecture based on three parts: a docking site offering specificity for the kinase of interest, a nuclear localization sequence that is inactivated by phosphorylation, and a fluorescent protein that reports on the nuclear-to-cytoplasm shuttling of the sensor. Herein, we demonstrate the modularity of this strategy by engineering sensors for the mating pathway MAPKs Fus3 and Kss1 as well as for the CWI MAPK Mpk1. These sensors allowed measurement of the dynamics of MAPK activation in single cells. We could identify a heterogeneity in the response of the cells upon α-factor or zymolyase treatments which demonstrates the ability of the MAPK signaling pathways to integrate intra-cellular cues to tune the response delivered by the MAPK.

## Results

### Sensor design

**Fig. 1:**
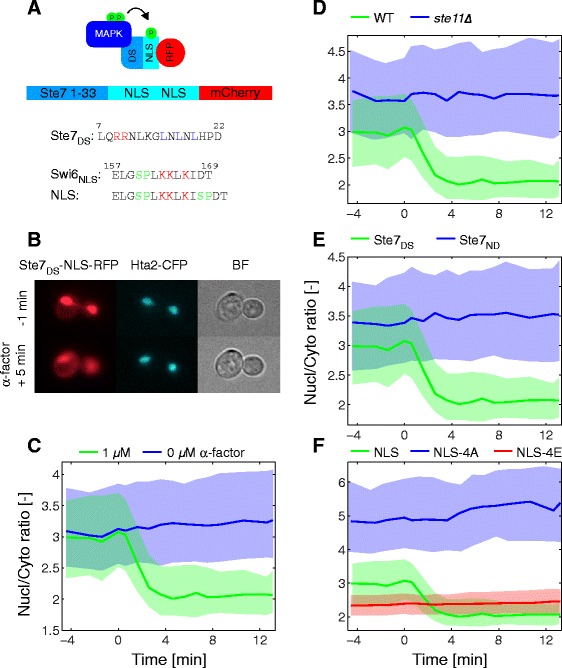
SKARS relocation depends on an intact docking site and phosphorylation of NLS by MAPK. **a** Schematic of the three domains of the SKARS sensor: the docking site (DS), the nuclear localization signal (NLS), and the fluorescent protein (RFP). The Ste7_DS_-NLS-RFP SKARS is composed of the Ste7 docking site (Ste7_DS_, amino acids 1-33), a double NLS, and the mCherry protein. **b** Microscopy images of cells before and after stimulation with α-factor. In the red channel, one can observe the nuclear exit of the sensor after stimulus while the CFP histone tag signal remains stable. **c–f** After quantification of the time-lapse movies, the ratio of nuclear-to-cytoplasmic fluorescence is plotted as function of time. The response of cells bearing the functional sensor and stimulated with 1 μM α-factor (Nc = 170, same curve on panels **c** to **f**) is compared to unstimulated cells (Nc = 550, **c**) or to cells deficient for signal transduction (*ste11∆*, Nc = 360, **d**), cells bearing a non-docking variant of the sensor (Ste7_ND_, Nc = 300, **e**), or a non-phosphorylatable (NLS-4A, Nc = 360, **f**) or phospho-mimicking variant of the sensor (NLS-4E, Nc = 750, **f**). Unitless measurements, such as the Nucl/Cyto ratio, are denoted by the symbol [–] in the axis legend. For all similar graphs and unless stated otherwise, the solid lines represent the median of the cell population and the shaded area the 25 and 75 percentile of the population. Nc represents the number of single cells measured

Nuclear localization sequences (NLSs) consist of a stretch of positively charged residues associated with importin, which will subsequently shuttle its cargo into the nucleus [14]. Harreman et al. [15] demonstrated that additional negative charges around the NLS compromise its binding to importin and thereby decrease the enrichment of the cargo in the nucleus. Further, they observed that the NLS for the Swi6 transcription cofactor was led by a serine, which is a potential phosphorylation target. Indeed, mutation of this residue to an alanine resulted in a constitutively nuclear Swi6 protein. Based on these findings, we used the Swi6 NLS as a phosphorylation target for our sensor, with the idea that phosphorylation would lead to an exit of the sensor from the nucleus. To increase the efficiency of relocation, we added a second phosphorylation site after the positive stretch of amino acids and combined two such NLSs in our sensor (Fig. 1a).

All MAPKs share the same consensus phosphorylation motif SP or TP. Specificity is achieved via an interaction surface in the kinase called the docking groove. This motif associates with a docking site (DS) with the consensus motif (R/K)_1-2_-X_4-6_-LXL, which has been identified on MAPK substrates as well as on MAPK phosphatases and up-stream activators (MAP2K) [16–18]. These interaction motifs are present throughout eukaryotes from yeast to mammals. Exchange of this DS has been shown to dictate specificity towards a substrate or, in the case of a MAP2K, control the specificity of the activated MAPK [19, 20]. The group of Wendell Lim has characterized various DSs that interact with Fus3 and Kss1 [20]. We used the first 33 amino acids of Ste7, which contains the DS with the strongest affinity towards Fus3 and Kss1. The Ste7_DS_ and double-NLS construct was fused to mCherry (Fig. 1a). As shown in Fig. 1b, the sensor is enriched in the nucleus under vegetative growth conditions. Upon stimulation of the cells with α-factor, the fluorescent protein quickly relocates to the cytoplasm. Automated segmentation of the images allows the identification of the nucleus and cytoplasm for each cell (Additional file 1: Figure S2) [21]. The ratio of the average intensities in the nucleus and in the cytoplasm is calculated as a function of time for each cell. The median, 25-, and 75-percentiles of the population are plotted for cells treated with 1 μM α-factor or unstimulated cells (Fig. 1c). At the onset of the experiment, the ratio is high, denoting an enrichment of the sensor in the nucleus. Upon α-factor addition, this ratio drops within a few minutes. This result suggests that the sensor is phosphorylated by Fus3 and Kss1 in response to α-factor treatment, leading to its relocation from the nucleus to the cytoplasm.

To demonstrate the specificity of this response, we repeated this experiment in cells deficient for MAPK activation (*ste11∆*), in which the sensor remained enriched in the nucleus upon stimulation (Fig. 1d). We can further demonstrate that this response depends on an intact DS. Indeed, mutation of the DS of Ste7 abolishes the response (Fig. 1e). Moreover, mutation of the four serines in the two NLSs to phospho-mimicking residues (glutamic acid: NLS-4E) or to non-phosphorylatable residues (alanine: NLS-4A) alters the localization of the fluorescent protein in the cell, but neither of these constructs display a change in nuclear-to-cytoplasmic partitioning upon pheromone stimulation (Fig. 1f).

**Fig. 2:**
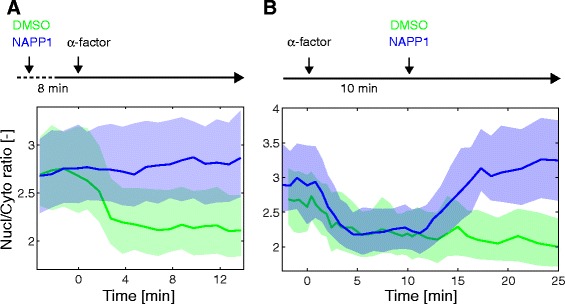
Inhibition of the MAPK Fus3 prevents the relocation of the sensor. *fus3∆kss1∆* cells bearing the Ste7_DS_-NLS-RFP SKARS and an integrated *fus3-as* were stimulated with pheromone in presence or absence of the inhibitor NAPP1. **a** NAPP1 or DMSO were added 8 minutes prior to the addition of α-factor. The median nuclear-to-cytoplasmic ratio is plotted as function of time (NAPP1: Nc = 343, DMSO: Nc = 230). **b** Ten minutes after α-factor stimulation, cells were treated with NAPP1 or DMSO. Note that, upon inhibition of Fus3-as, the sensor returns within 7 minutes to its basal nuclear enrichment level (NAPP1: Nc = 177, DMSO: Nc = 123)

Finally, we also verified that the direct inhibition of the MAPK using an analog-sensitive allele blocked the phosphorylation and hence the relocation of the SKARS (*fus3-as* in *fus3∆kss1∆*, Fig. 2a) [22]. Interestingly, addition of the inhibitor after the activation of the pathway leads to a rapid return of the sensor to its basal nuclear enrichment level, underlying the dynamic nature of the biosensor phosphorylation (Fig. 2b). Taken together, these results clearly demonstrate that we have generated a specific sensor for Fus3 and Kss1 activity. The relocation is dependent on an active kinase that binds to the sensor via the specific DS and phosphorylates the serines in the NLSs. This modification is reversible, allowing for a dynamic measurement of the MAPK activity.

To assess if the presence of the SKARS perturbs the mating response of the cells, we confirmed that cells could arrest their cell cycle and form mating projections (Additional file 1: Figure S3A and B). These qualitative tests revealed no difference between cells bearing docking or non-docking versions of the sensor. However, we noted a minor difference in the transcriptional response of cells due to the presence of the sensor by quantifying by flow cytometry the expression of the p*FIG1*-quadrupleVenus expression reporter [23] (Additional file 1: Figure S3C). This slight increase in expression could potentially be explained by a minor enrichment of the MAPKs in the nucleus induced by the presence of the sensor. However, this artefact should not prevent a faithful measurement of the dynamics of MAPK activity.

### Inferring MAPK activity

**Fig. 3:**
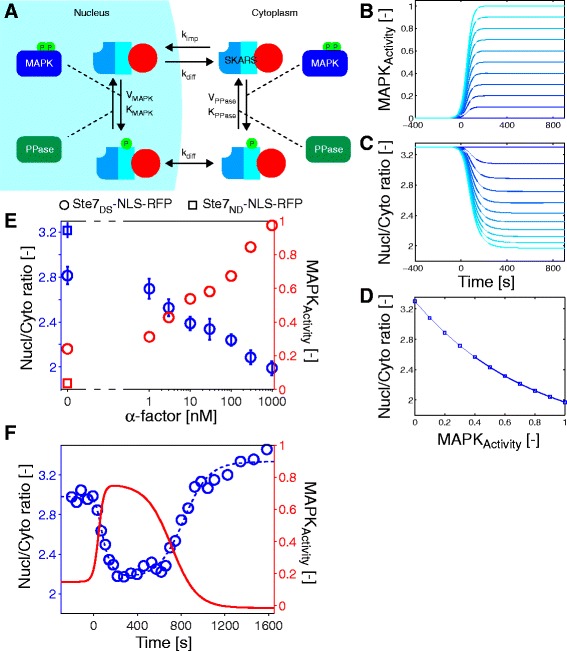
Determination of MAPK activity from biosensor measurements. **a** Schematic of the reactions implemented in the model. The SKARS is phosphorylated in the nucleus and in the cytoplasm by MAPK and dephosphorylated by phosphatase. The exchange of SKARS between the cytoplasm and the nucleus can occur through passive diffusion, while only the unphosphorylated SKARS is actively imported in the nucleus. **b, c** Time-course of activity of MAPK is calculated for different final level of MAPK activity and used as inputs into the model (**b**). The resulting nuclear-to-cytoplasmic partitioning of the sensor for these different MAPK activity traces are obtained as output (**c**). **d** Correlation between MAPK activity and the nuclear-to-cytoplasmic ratio at the end of the simulation. **e** Dose-response curve of the nuclear enrichment of the sensor as a function of concentration after 15 minutes of stimulation with pheromone (blue circles). The mean and standard deviation of three measurements are plotted. The corresponding MAPK activity level is calculated (red circles). As reference, the nuclear-to-cytoplasmic ratio of the unstimulated non-docking sensor and its corresponding level of MAPK activity are indicated by blue and red squares. **f** Determination of MAPK activity for the experiment from Fig. 2b. The blue circles correspond to the experimental data points. The dashed blue line represents the fit of those points by the model. The solid red line is the extrapolated MAPK activity for this time-course

To extract MAPK activity from sensor relocation measurements, we have developed a simple mathematical model describing the relationship between the relative concentration of the sensor in the nucleus and in the cytoplasm and the activity of MAPK. Figure 3a is a scheme of the various reactions included in the model (Additional file 1: Supplementary Text and Table S1 and S2). A MAPK and a phosphatase regulate the level of phosphorylation of the sensor. The action of the phosphatase is constitutive. The concentration of MAPK in the cell is constant; however, the ratio of active MAPK (MAPK_Activity_ defined as the fraction of the total MAPK pool in the active state) changes as a function of time. For simplicity, we consider that these reactions occur with similar rate constants in the nucleus and in the cytoplasm. The sensor can freely diffuse between the nucleus and the cytoplasm (k_Diff_), but only the unphosphorylated SKARS is actively imported in the nucleus (k_Imp_). The model can be forward-simulated by generating a specific time-course of MAPK_Activity_ (Fig. 3b) and calculating the resulting nuclear-to-cytoplasmic partitioning of the sensor (Fig. 3c). At steady-state, there is a close to linear relationship between the MAPK_Activity_ and the nuclear-to-cytoplasmic ratio of the sensor (Fig. 3d).

We can also use the model to estimate MAPK activity based on a measured nuclear-to-cytoplasmic ratio. We quantified the steady-state nuclear enrichment of the sensor in a population of cells 15 minutes after induction with various concentrations of α-factor (Fig. 3e). Based on these data, we can obtain the dose response curve of MAPK_Activity_, which extends from 0.2 for an unstimulated sample to 1 for saturating levels of pheromone (1 μM). A MAPK_Activity_ of 1 implies that both Fus3 and Kss1 are completely phosphorylated in saturating levels of pheromone. While this level might seem high, a recent study on the HOG pathway suggests that close to 100 % of MAPK Hog1 is phosphorylated upon hyper-osmotic shock [24]. A non-docking mutant is used as reference and displays no MAPK_Activity_. It has been shown that HOG and the mating pathways have some basal level of MAPK activity under normal growth conditions [25]. The exact level of this basal signal is difficult to estimate, but we consistently observe an increase in nuclear enrichment of the sensor in signaling dead cells as well as in non-docking versions of the sensor.

Using a similar strategy, the dynamics of MAPK activity can be estimated from time-course measurements of sensor relocation. As a proof a concept, we use the activation and inhibition of the pathway by α-factor and subsequent inhibitor addition presented in Fig. 2b. Before stimulus, the MAPK_Activity_ was low. Upon α-factor addition, it reached 0.8 in less than 3 minutes after stimulus. Upon inhibition, MAPK_Activity_ dropped at a much slower rate. This decay is controlled by multiple factors such as the time of entry of the drug in the cell, the dephosphorylation of the biosensor, and its exit by diffusion out of the nucleus. This mathematical model demonstrates that there is a tight relationship between SKARS localization and MAPK activity and that, using a few simple assumptions, it is possible to extract the mean MAPK activity of the population from the experimental measurements.

### Single cell measurements

While a clear exit of the sensor out of the nucleus can be measured at the population level, we next wanted to verify whether we could also obtain kinase activity measurements at the single cell level. We noticed that there is a large variability in the nuclear enrichment of the sensor between individual cells prior to induction (Additional file 1: Figure S4A). This large difference could arise from varying basal activity of the pathway [25] or from an intrinsic ability of a given cell to accumulate the sensor in the nucleus. To differentiate between these two possibilities, we combined a functional (Ste7_DS_-NLS-YFP) and a non-functional sensor (Ste7_ND_-NLS-RFP) in the same cell. We define the Basal Level as the nuclear-to-cytoplasmic ratio of the SKARS before induction (Additional file 1: Figure S4A). Using this measurement, we observe a high correlation between the nuclear enrichment of the two sensors within a cell, which demonstrates that each cell has an inherent ability to import the sensor (Additional file 1: Figure S4B). Evidently, in cells displaying a higher nuclear enrichment of the sensor, the change in nuclear-to-cytoplasmic ratio upon stimulation is easier to quantify (Additional file 1: Figure S4A). To allow for a fair comparison between each individual single cell trace, they were normalized by their basal levels. For each individual trace, the initial response (3 to 5 minutes after stimulus) and the final response (last three points of the time-lapse) were quantified (Additional file 1: Figure S4C to E).

**Fig. 4:**
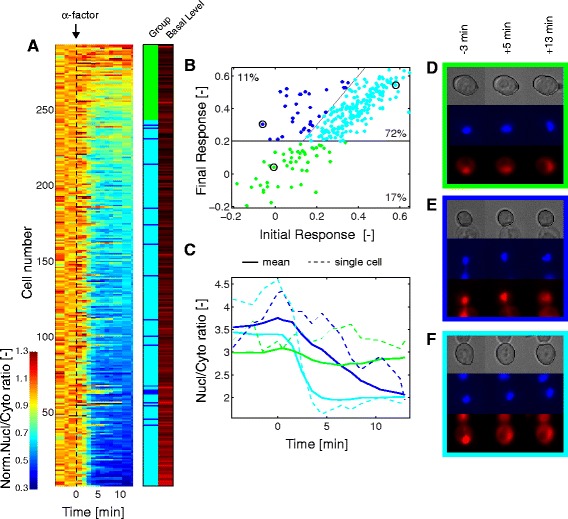
Single cell analysis of MAPK activity upon pheromone stimulation. **a** Heat map of the response of individual cells to 1 μM α-factor. Each line represents the nuclear-to-cytoplasmic ratio of one single cell normalized by its basal level (Nc = 293). The cell responses were sorted based on the level of final response (measured at T = 12 min). The cells were classified as responding (cyan), non-responding (green), or slow responding (blue). The basal level of each trace is indicated in shades of red (from black to red, increasing basal level). **b** Scatter plot of initial and final response of the cells. Each dot corresponds to a single cell measurement. The colour indicates to which sub-population the cells belong to. **c** Temporal evolution of the nuclear-to-cytoplasmic ratio upon pheromone treatment. The solid line represents the mean response of each sub-population. The dashed lines are traces from the three single cells circled in panel (**b**). **d**, **e**, **f** Images of the selected cells in the brightfield, CFP (histone tag), and RFP (Ste7_DS_-NLS-RFP) channels at different time points. The dark line in the brightfield image represents the segmented cell contour

The normalized nuclear-to-cytoplasmic ratio of roughly 300 single cells were sorted based on their final responses and displayed in a heat map where each line corresponds to a single cell trace (Fig. 4a). In the lower part of the map, cells displaying a large change in nuclear-to-cytoplasmic enrichment upon stimulation are clustered (high final response). The upper part of the map represents a fraction of the population where no response is detected. To discriminate the responding cells from the non-responding ones, we compared the single cell responses of two strains bearing either the docking or the non-docking sensor (Ste7_DS_-NLS-RFP and Ste7_ND_-NLS-RFP, respectively). Using the non-functional sensor as a reference, we set a threshold to discriminate between the responding cells and non-responding cells bearing the functional sensor (Additional file 1: Figure S4F to H). Roughly 17 % of the cells measured were thus characterized as non-responding (Fig. 4b).

In the group of responding cells, we could also differentiate two types of responses. A small fraction (11 %) of the total cell population displayed a delayed response, characterized by an initial response weaker than the final response (arbitrarily defined as more than a 50 %-fold difference, Fig. 4b). Figure 4c represents the mean response of the cells in the defined categories (solid lines) as well as the response of one cell from each sub-population (dotted line), identified by a circle in panel B. Images of these same cells are also provided in panels D, E, and F. The heterogeneity in the dynamics of SKARS between these three populations of cells suggests that they have very different levels of MAPK activity, which may be linked to an intrinsic difference in their ability to respond to mating pheromone.

**Fig. 5:**
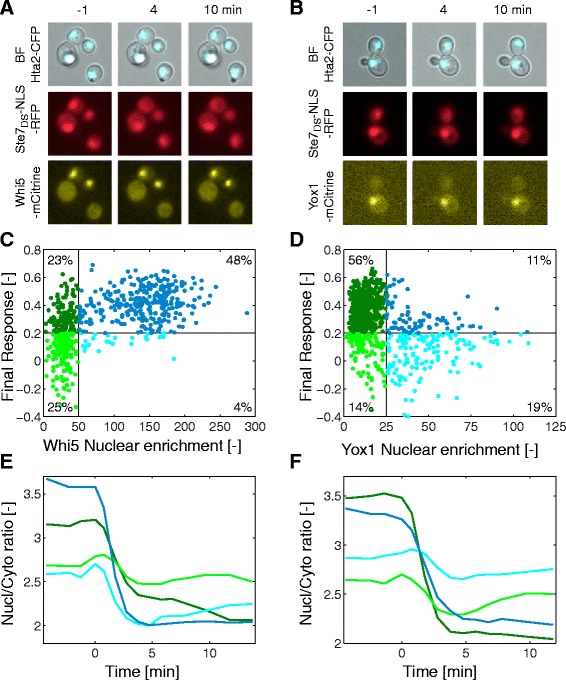
Cell cycle dependent dynamics of signaling in the mating pathway. **a, b** Yeast cells expressing the Ste7_DS_-NLS-RFP sensor and Whi5 tagged with mCitrine (**a**) or Yox-1 tagged with mCitrine (**b**) imaged before and after stimulation with α-factor. **c**, **d** Correlation between the measured Whi5 (Nc = 616, **c**) and Yox1 (Nc = 698, **d**) nuclear enrichment (nuclear – cytoplasmic intensities) measured before stimulation and the sensor’s final response. The scatter plot is split in four quadrants. The blue dots represent cells with an enriched nuclear marker, while the green dots represent cells where no enrichment is detected. The dark and light coloured dots represent, respectively, responding and non-responding cells. **e, f** Average response of the four sub-populations of cells in the four quadrants delimited on panels **c** (Whi5, **e**) and **d** (Yox1, **f**). The colour code is the same as in (**c**) and (**d**)

It is indeed well-established that the signaling activity in the mating pathway is dependent on the cell cycle stage [26–28]. Cells which are committed to division and have entered the S-phase become refractory to signal transduction. To correlate cell cycle stage with the dynamics of the SKARS, we tagged Whi5 and Yox1 with mCitrine in cells bearing the sensor. Whi5 is a repressor of G1 transcription and accumulates in the nucleus of the cells during G1 (Fig. 5a). It has been used previously as a marker for G1-entry and exit [29]. Yox1 is a transcription repressor that inhibits the expression of cell cycle regulated genes induced in the M and G1 phases [30]. It enters the nucleus upon S-phase entry and remains there until the beginning of G2 (Fig. 5b). As expected, cells with nuclear Whi5 were predominantly responding (Fig. 5c, dark blue dots). In contrast, a large fraction of the Yox1 positive cells were non-responding (Fig. 5d, light blue dots). Cells with cytoplasmic Whi5 were equally split between non-responding and responding cells. Based on the Yox1 data, we can assume that these non-responding cells are mainly S-phase cells (4C, light green dots) and, therefore, the responding ones are G2/M cells (4C, dark green dots). Similarly, we can assume that the Yox1-negative population, which displays a response, is dominated by G1 cells (4D, dark green dots), while responding cells with a Yox1-positive signal must be in the G2 phase (4D, dark blue dots).

Taken together, these results are in general agreement to what was previously known: G1, M, and G2 cells are permissive to α-factor signaling while S-phase cells are refractory to this stimulus. However, these data also offer some additional insights about this regulation. First of all, there is a clear kinetic difference in the activation of the sensor between G1 cells and G2/M cells. In G1 cells, the sensor exits the nucleus within 3 to 4 minutes after the stimulus, while the G2/M cells seem to display a slower response (Fig. 5e, dark blue vs. dark green curves). This sub-population of cells is clearly enriched for slow responding cells (Additional file 1: Figure S5). This suggests that Fus3/Kss1 activity in this phase of the cell cycle is reduced. The second interesting observation is the fact that almost all sub-populations display an early exit of the sensor, which subsequently returns to basal value for non-responding cells (Fig. 5e, light green curve and 5f light blue curve). This hints at a transient activation of the pathway at early time-points, which cannot be sustained if the cells are not in the proper cell cycle stage.

### Combination of sensors

**Fig. 6:**
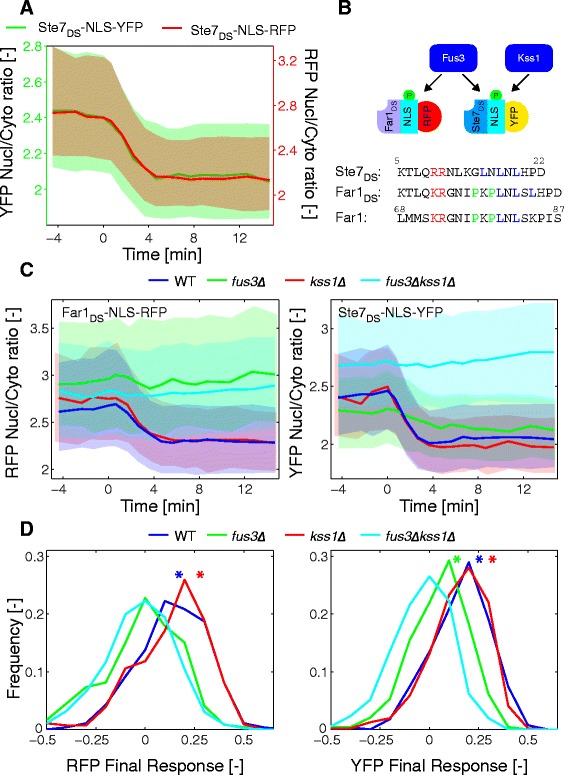
Generation of a Fus3-specific sensor based on the Far1 docking site. **a** Combination in the same cell of two sensors bearing the same Ste7_DS_ but tagged with mCherry or mCitrine and stimulated at time 0 with 1 μM α-factor (Nc = 660). The ratios were plotted on two different y-axes (left: Ste7_DS_-NLS-YFP, right: Ste7_DS_-NLS-RFP) to allow a direct comparison of the dynamic response of both sensors. **b** Engineering of a Fus3-specific sensor by replacing the Ste7 docking site by the Far1 docking site sequence (Far1_DS_). **c** Response of cells of different background (WT (blue, Nc = 630), *fus3∆* (green, Nc = 474), *kss1∆* (red, Nc = 990), and *fus3∆kss1∆* (cyan, Nc = 952)) bearing the Far1_DS_-NLS-RFP (left) and the Ste7_DS_-NLS-YFP (right) stimulated with α-factor 1 μM. **d** Histograms of the final response of cells in the YFP and RFP channel. The asterisks designate distributions that are significantly different (*t*-test, *P* <0.001) from the non-responding *fus3∆kss1∆* control

In the previous experiment, SKARS were associated with fluorescently tagged proteins to correlate kinase activity and cell cycle stage. In a similar manner, we can envision to combine multiple SKARS in the same cell to correlate the activity of different kinases. Indeed, the fluorescent protein in the sensor is an inert bystander of the relocation process and its exchange should not affect the response of the sensor. To verify this statement, we combined an RFP and a YFP version of the sensor (Ste7_DS_-NLS-RFP and Ste7_DS_-NLS-YFP) in the same strain. While there is a slight difference between the YFP and RFP ratio measured with the sensor, the dynamics of the response measured with both sensors are strikingly similar (Fig. 6a). At the single cell level, we measured a high correlation between the final responses measured with each sensor (Additional file 1: Figure S6A).

The Ste7 DS allows the sensing of the combined activity of Fus3 and Kss1. In their analysis of MAPKs DSs, Lim *et al*. [20] revealed the difference in structure between the DS of Ste7, which binds both Fus3 and Kss1, and the DS of Far1 that is specific for Fus3. The presence of two prolines in the Far1_DS_ forces binding to MAPK via a different conformation that is not compatible with the Kss1 docking groove. To test whether we could confer Fus3 specificity to our sensor, we replaced the 11 amino acids in the Ste7_DS_ by the 13 residues forming the Far1_DS_ (Fig. 6b). To test the specificity of this sensor, we combined the Far1_DS_-NLS-RFP and Ste7_DS_-NLS-YFP sensors in the same cells and quantified their responses in wild-type (WT) and MAPK deletion strains (Fig. 6c and d). Both sensors relocate in WT and *kss1∆* backgrounds. As expected, all relocation is abolished in the double MAPK deletions *fus3∆kss1∆.* Interestingly, in *fus3∆* cells, only the Ste7_DS_-NLS-YFP sensor exits the nucleus upon α-factor stimulation, demonstrating the specificity of the Far1_DS_ for phosphorylation by the Fus3 MAPK. The histogram of the final response measured in individual cells is displayed in Fig. 6d. In *fus3∆* cells, a weak but statistically significant activation of the pathway can be detected. Note also that the basal nuclear enrichment of the Ste7_DS_-NLS-YFP sensor in this strain is lower, which suggests that, in the absence of Fus3, there is a slightly higher level of MAPK activity. It is in agreement with previous observations showing that Kss1 is overexpressed and displays a higher basal activity in a *fus3∆* background [31]. It is noteworthy to mention that both the dynamics and level of kinase activity in WT and *kss1∆* cells are very similar, arguing for a predominant contribution of Fus3 to the response of the cells when stimulated with 1 μM α-factor.

### Cell wall integrity pathway

**Fig. 7:**
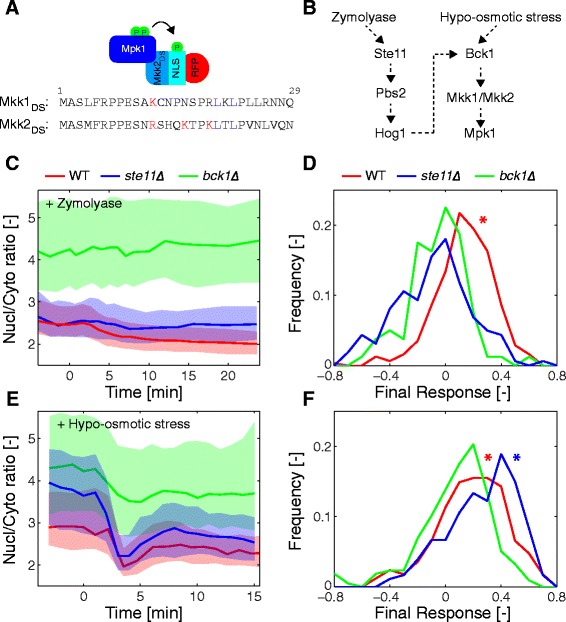
Mpk1 activity dynamics upon zymolyase or hypo-osmotic stresses. **a** Schematic of the Mpk1 sensor and alignment of the docking sites present in Mkk1 and Mkk2. **b** Scheme of the activation of Mpk1 by zymolyase or hypo-osmotic stress. **c** Dynamic localization of the Mkk2_DS1-100_-NLS-RFP sensor quantified by its change in nuclear-to-cytoplasmic ratio upon stimulation by zymolyase 3 U/mL at time 0 in WT (red, Nc = 313), *ste11∆* (blue, Nc = 160), and *bck1∆* (green, Nc = 80). **d** Histograms of the final response after zymolyase stress measured in the WT and two deletion strains. **e** Mpk1 sensor relocation upon hypo-osmotic shock performed in microfluidic devices where the sorbitol concentration in the medium is lowered from 1 M to 0.5 M: WT (red, Nc = 168), *ste11∆* (blue, Nc = 180), and *bck1∆* (green, Nc = 217). **f** Histograms of the final response after hypo-osmotic stress measured in the WT and two deletion strains. In panels D and F the asterisks designate distributions that are significantly different (*t*-test, *P* <0.001) from the *bck1∆* control

Since the DS of the sensor dictates its specificity, we next tried to identify a DS for the MAPK of the CWI pathway, Mpk1. Molina et al. [32] identified a sequence in the N-terminus of the MAP2Ks Mkk1 and Mkk2 of *S. cerevisiae* that is conserved in other fungi and share some homology with the consensus DS of MAPKs (Fig. 7a). We therefore cloned the first 33 amino acids of Mkk2 in front of the NLS-RFP construct to generate an Mpk1-specific SKARS. When integrated in cells, this sensor turned out to be mostly cytoplasmic, suggesting that a high basal activity of the MAPK resulted in a constitutive phosphorylation of the sensor. Indeed, this localization was Mpk1 activity dependent since deletion of the MAP3K Bck1 leads to an enrichment of the sensor in the nucleus (Additional file 1: Figure S7A and B).

To modulate the sensitivity of the sensor, we changed the distance separating the DS and the NLS. Mkk2 N-terminal fragments of different lengths (from 1–27 to 1–150 amino acids) were cloned in front of the NLS hoping that the distance between the DS and the phosphorylation sites would tune the ability of Mpk1 to phosphorylate the sensor. While we did not detect a simple relationship between the length of the spacer and the basal level of nuclear localization of the SKARS (Additional file 1: Figure S7C to F), we could identify one construct, DS_1-100_, which displayed the highest enrichment in nuclear fluorescence. Nonetheless, the nuclear enrichment of this SKARS was still highly variable from cell to cell, suggesting that cells within a population can display a wide range of Mpk1 activities.

To test whether the Mkk2_DS1-100_ sensor could report on Mpk1 activation by acute cell wall stress, cells were subjected to zymolyase treatment. It has been previously reported that in order to activate Mpk1 upon zymolyase stress, activity of the HOG pathway via Ste11 and Pbs2 is required (Fig. 7b) [6]. Due to the very large difference in basal value between *bck1∆* cells on one side and WT and s*te11∆* on the other, the latter strain offered a better comparison for the response measured in WT cells (Fig. 7c). When measured at the population level, the WT cells stressed with zymolyase displayed a slow gradual relocation of the sensor out of the nucleus. As expected, this translocation was blocked in *bck1∆* as well as in *ste11∆*, implying that Mpk1 was not activated in these mutants (Fig. 7d).

In order to verify that the response of the SKARS was specific to the Mpk1 pathway, we also stressed cells with hypo-osmotic shock, where CWI activation is independent of the HOG pathway (Fig. 7b). To perform this experiment, cells were grown in medium complemented with 1 M sorbitol. The cells were loaded in a microfluidic chip and the medium was exchanged within seconds by medium containing 0.5 M sorbitol. The sensor transiently exited the nucleus and returned to a stable value slightly lower than the original basal value. Ste11 was not required for this response, as the *ste11∆* cells displayed a response comparable to the WT cells. In *bck1∆* cells, only a small drop in the nuclear-to-cytoplasmic ratio was detected corresponding to the expansion of the cell in the lower osmolarity medium (Fig. 7e,f). Note also that the sensor was more nuclear in cells grown in 1 M sorbitol, implying that the basal activity of the pathway decreased under these conditions and that the deletion of Ste11 also influenced this basal activity in hyper-osmotic medium.

### Single cell response to cell wall damage

**Fig. 8:**
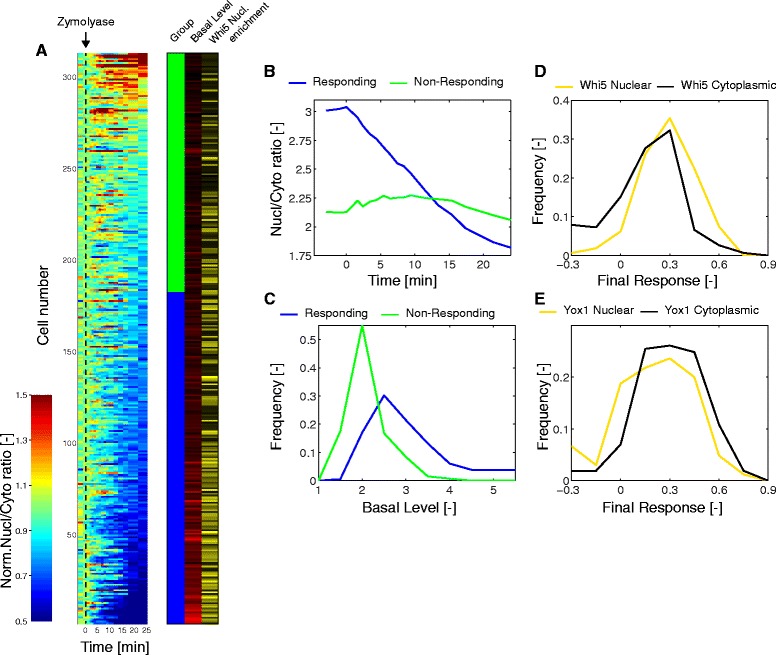
Single cell responses upon zymolyase-induced cell wall damage. **a** Heat map of the response of individual cells bearing the SKARS Mkk2_DS1-100_-NLS-RFP to zymolyase (3 U/mL). Each line represents the normalized response of one single cell (Nc = 313). The cell traces were sorted based on the level of final response. This measurement was used to classify the cells in a group of responding cells (blue) and a group of non-responding cells (green). The basal level of each trace is indicated in shades of red (from black to red, increasing basal level). These cells also bear the cell cycle marker Whi5 and its level of nuclear enrichment is indicated in shades of yellow (from black to yellow, increasing nuclear Whi5). **b** Average nuclear-to-cytoplasmic ratio as function of time for the responding and non-responding cells. **c** Histogram of the basal level in responding and non-responding cells. **d, e** Histogram of the final response measured in cells with or without nuclear enrichment of the cell-cycle marker Whi5 (**d**) or Yox1 (**e**).

Because of the wide variety in basal activity of Mpk1 observed in the cell population, we wondered how individual cells responded to zymolyase treatment. Figure 8a represents a 2D-map of the normalized response of more than 300 single cells sorted based on their final response. Using a threshold of 0.2 for the final response, we can split the population in a group of responding (58 %, blue) and non-responding cells (green). Figure 8b displays the average nuclear-to-cytoplasmic ratio of the sensor as function of time for these two populations. The non-responding cells have a strikingly lower level of sensor nuclear enrichment at the onset of the experiment denoting a higher basal activity of the pathway in these cells (Fig. 8c).

Since it has been reported that the activity of the Mpk1 pathway fluctuates during the cell cycle and is higher in phases of polarized growth, we wondered whether the heterogeneity observed in the response to zymolyase was dependent on cell cycle stage [33]. G1 cells in the population were identified with a Whi5-mCitrine tag. The level of nuclear accumulation of Whi5 for each cell is shown in Figure 8a; cells with nuclear Whi5 can be found in both the responding and non-responding cells. Figure 8d compares the final response of cells with nuclear and cytoplasmic Whi5. A similar analysis was performed with Yox1-mCitrine cells, where the presence of the fluorescent protein in the nucleus identifies S-phase and early G2-phase cells. There was a tendency for G1 cells to respond stronger to the stimulus while dividing cells displayed a decreased tendency to respond. However, there was no apparent inhibition of CWI signaling in a cell cycle-dependent manner.

To modulate the basal activity of the pathway, we grew cells in low glucose (0.01 %, Additional file 1: Figure S8). In this medium, the growth rate was slower, thus remodelling of the cell wall occurred on longer time-scales, resulting in a lower constitutive basal activity of Mpk1. Indeed, we observed a global increase in the basal nuclear enrichment of the sensor, resulting from the decreased activity of Mpk1 (Additional file 1: Figure S8B). A larger fraction of the cells stressed with zymolyase under these conditions displayed an export of the sensor (Additional file 1: Figure S8C). Together, these results indicate that basal activity of the CWI pathway is linked with cell growth rather than the cell cycle. Moreover, in cells where Mpk1 is already activated by growth, we do not detect an additional effect due to the cell wall stress induced by zymolyase within the time-frame of the experiment.

## Discussion

In this paper, we demonstrate the use of a new set of biosensors to measure the fast dynamics of signal transduction in living single cells. The functional part of the sensor is a double NLS sequence bearing four phosphorylation sites, which results in a three-fold enrichment of the reporter in the nucleus of the cells under basal conditions. This is, however, not a static situation and the sensor constantly shuttles back and forth between the cytoplasm and the nucleus. The import into the nucleus is actively driven by the NLSs while the exit out of the nucleus is a passive mechanism relying on diffusion due to the small molecular weight of the sensor [34]. Upon phosphorylation of the NLS, the import rate into the nucleus will be decreased due to a lower affinity for importin, while the diffusion rate remains the same, resulting in a lower concentration of the nuclear sensor. Note that phosphorylation can occur in both compartments due to the constant exchange between the two sensor pools. Therefore, measurement of kinase activity is not restricted to one of these compartments, and the sensor measures the global activity of the enzyme in the cell. Moreover, using a simple mathematical model, it is possible to infer the amount of MAPK activity from the SKARS measurements.

The action of the kinase on the sensor is antagonized by phosphatases. These dephosphorylation events occur on the minute time-scale, as attested by our inhibition of Fus3 experiment (Fig. 2). Since the signal transduction event in the MAPK cascade occurs on a similar time-scale, the SKARS are perfectly suitable to record these fast signaling events. Our sensor ultimately measures the balance between kinase and phosphatase activities on the phosphorylatable residues on the NLS. In MAPK pathways, signaling results in a change in the activation of the MAPKs while the phosphatases possess a relatively stable and constitutive activity. Part of the variability observed with our sensor could possibly arise from differences in phosphatase activity between individual cells.

Förster Resonance Energy Transfer (FRET) probes have been generated to monitor the activity of kinases in cells [12, 35, 36]. In comparison, the SKARS possesses two main advantages. First, the requirement of the FRET probe to use two fluorophores together with the limited number of available FRET pairs makes it difficult to combine two different FRET sensors in the same cell. In comparison, our system uses a single fluorescent protein and we have showed the correlated measurements of two reporters in the same cell. This number could be increased to three or four by combining different fluorescent protein spectral variants [37].

The second advantage of our system lies in it is modularity. The optimization of the FRET sensor is a difficult task because the orientation of the two fluorophores has to be adjusted and depends directly on the structure of the sensor’s active site [38, 39]. In the SKARS, the substrate site and the fluorescent protein are independent of each other, and can therefore be interchanged without affecting each other. Indeed, the fluorescent moiety was exchanged between mCherry and mCitrine without affecting the dynamics of the response. Similarly, we could exchange the Ste7 DS with a Far1 DS or Mkk2 DS to confer specificity of the sensor towards Fus3 or Mpk1, respectively. We also demonstrated that the sensitivity of the sensor can be tuned by changing the distance between the DS and the phosphorylatable residues in the NLS.

Interestingly, NLSs as well as MAPK DSs are conserved domains throughout all eukaryotes. This implies that a similar strategy can be adopted to develop sensors for higher eukaryotes. In fact, other kinase sensors based on phosphorylatable NLS or nuclear exclusion sequences have been described in the literature both in yeast and in higher eukaryotes by using small domains of proteins known to relocalize [40, 41]. In parallel to the development of our biosensor, Regot et al. [42] have used a similar strategy to quantify MAPK activity in mammalian cells. This underscores the wide applicability of the approach presented here. In this work, we used a synthetic biology approach to design *de novo* a MAPK substrate that can undergo relocation. Using a similar strategy, we can also envision measuring other enzymatic activities in living cells. Extending the range of available SKARS for other kinases should be relatively straightforward if they require a DS and possess a short consensus motif. Other kinases with longer consensus phosphorylation sites could also be monitored provided that phosphorylatable residues can be placed close enough to the NLS to influence its association with the importin.

## Conclusions

Using these biosensors to probe MAPK activity in the mating and cell wall integrity pathways, we could reveal a large heterogeneity in the response between cells within an isogenic population. For the mating pathway, this variability can be explained by extrinsic differences between cells. While it has been known that the response to mating pheromone was cell cycle-dependent with an inhibition of the pathway during the S phase, we could demonstrate with these sensors that there is also a different kinetic activation of the pathway between a fraction of G2/M cells and cells in the G1 phase. In the S phase, the scaffold protein Ste5 is phosphorylated by the Cln2/Cdc28 complex to prevent its recruitment at the plasma membrane via the PM domain [28]. It remains to be seen whether the same mechanism is involved in the observed difference in the kinetics between G1 and G2/M cells. In the CWI pathway, a heterogeneity in the response can also be observed between individual cells. It cannot be attributed to cell cycle-specific cues, but rather to the general growth of cells that induce more or less basal activity of the Mpk1 MAPK.

These two examples confirm the notion that MAPK pathways are not isolated signal transduction cascades, but are embedded in the global signaling network of the cell. MAPK pathways can integrate these intra-cellular signals to modulate their output, and in turn the MAPK activity can impact on cellular processes such as the cell cycle, metabolism, or growth. The ability to measure MAPK activity in single cells provided by the SKARS enables us to uncover these regulations and provides the opportunity to identify the mechanistic links that connect MAPK pathways to other cellular processes. In general, the SKARS technology enables the direct measurements of enzymatic activity in living cells and as such can become an important tool for system biology. Its use will allow the identification of new regulatory mechanisms present in these signaling cascades and, in turn, will enable to build more accurate mathematical models of the dynamic events taking place in these pathways.

## Methods

### Strains and plasmids

Yeast strains and plasmids are listed in Additional file 1: Tables S4 and S5. SKARS plasmids were constructed by cloning the different parts of the sensor into a pRS304 or pRS306 vector backbone [43]. They were expressed from the constitutive promoter pRPS2, cloned SacI-HindIII. The DS from STE7, FAR1, and MKK2 were either amplified from yeast genomic DNA or synthetized and subsequently cloned between HindIII and XbaI. To generate the 2xNLS sequence, two oligos were synthetized, annealed, and cloned in the vector by enzymatic restriction with XbaI and BglII. The fluorescent protein mCherry or mCitrine (A206K L221K) were cloned between BglII and XhoI. A CYC1 terminator is cloned between XhoI and KpnI.

The plasmids were transformed in yeast from W303 background bearing an Hta2-CFP nuclear marker (ySP37). Genes were subsequently deleted in cells bearing the sensors using KAN or NAT resistance cassettes [44, 45]. Whi5 and Yox1 where tagged with mCitrine using the pKT139 plasmid [46].

### Sample preparation

The cells were grown overnight in synthetic medium to saturation (YNB: CYN3801, CSM: DCS0031, ForMedium). They were diluted to OD_600_ 0.05 in the morning and grown for at least 4 hours before the start of the experiment. For experiments in well slides, selected wells of 96-well plates (MGB096-1-2LG, Matrical Bioscience) were coated with a filtered solution of Concanavalin A in H_2_O (0.5 mg/mL, 17-0450-01, GE Healthcare) for 30 min, rinsed with H_2_O, and dried for at least 2 hours. Before the experiment, the cells were diluted to OD_600_ 0.05, and briefly sonicated before 200 μL of cells were added to each well. Imaging was started 15–30 minutes after to allow cells settle to the bottom of the wells. To stimulate the cells, 100 μL of inducing solution was added to each well. For α-factor stimulation, the inducing solution contained 3 μM of pheromone resulting in a 1 μM final concentration. Zymolyase (120491-1, AMS Biotechnology) stress was applied with a 9 U/mL concentrated solution resulting in a 3 U/mL stress. To inhibit Fus3-as (Fig. 2), a 25 mM stock solution of NAPP1 (A603004, Toronto Research Chemical) in DMSO was diluted in SD-medium to 40 μM. The solution was added to the well to obtain a final working concentration of 10 μM. In control experiments, cells were treated with DMSO 0.16 % in SD-full.

For the dose response experiments, the stimulation with pheromone was performed in Eppendof tubes. Specifically, 200 μL of cell culture were mixed with 100 μL of 3-fold concentrated α-factor to reach a final concentration spanning from 0 to 1 μM. Immediately after mixing, 200 μL of this suspension were loaded onto a coated 96-well plate. After 15 minutes of incubation, images from ten different fields of view were acquired.

For the hypo-osmotic stress experiment (Fig. 6e,f), cells were grown overnight in SD medium containing 1 M sorbitol, diluted in the morning in the same medium to OD_600_ 0.05, and grown for at least 4 hours before the start of the experiment. Cells were diluted to an OD_600_ of 0.25 if necessary and 200 μL of culture was loaded in a microfluidic chip (Y04C, CellASIC Corp). SD medium with 1 M sorbitol and containing fluorescein-dextran (D3305, Invitrogen) 1 μg/mL was added to wells 1 and 2. SD medium with 0.5 M sorbitol and containing fluorescein-dextran 0.5 μg/mL was added to wells 3 and 4. To allow for a rapid switching between the media, the pressure was set at 3 psi in wells 1 and 2 and at 0.3 psi in wells 3 and 4. At the desired time, the high and low pressures were inverted to allow entry of the low osmolarity medium into the cell imaging chamber.

### Microscopy

Images were acquired on a fully automated inverted epi-fluorescence microscope (Ti-Eclipse, Nikon) controlled by micro-manager [47] and placed in an incubation chamber set at 30 °C, with a 40× oil objective and appropriate excitation and emission filters. The excitation is provided by a solid-state light source (SpectraX, Lumencor). The images were recorded with an sCMOS camera (Flash4.0, Hamamatsu). A motorized XY-stage allowed recording multiple fields of view at every time point. CFP (40 ms), RFP (100 ms), and YFP (100 ms, when needed) and two brightfield (10 ms) images were recorded at time intervals varying from 1 to 5 minutes.

### Data analysis

Time-lapse movies were analyzed with the YeastQuant platform [21] (Additional file 1: Figure S2). The cell nuclei were segmented by thresholding of the CFP images. The contour of the cell around each nucleus was detected using two brightfield images. The cytoplasm object was obtained by removing the nucleus object expanded by two pixels from the cell object. The nuclei were tracked across all the frames of the movie. Multiple features of each object were quantified. Dedicated scripts in Matlab (The Mathworks) were written to further analyse the data. Only cells tracked from the beginning to the end of the movie were taken into consideration. In addition, for single cell analysis, a quality control was applied on each trace and only the traces with low variability in nuclear and cell area, CFP nuclear fluorescence, and RFP cellular fluorescence were kept for further analysis.

For each cell, the average nuclear intensity in the fluorescent channel corresponding to the SKARS was divided by the average intensity in the cytoplasm for every time point to generate the single cell trace. The basal level was calculated as the mean of the first three time points of the nuclear-to-cytoplasmic ratio. To obtain the normalized nuclear-to-cytoplasmic ratio, every time point was divided by the basal level. The final response was calculated as one minus the average of the last three time-points of the normalized nuclear-to-cytoplasmic ratio. The initial response was calculated as one minus the average of three time-points shortly after the stimulus (3–5 minutes after α-factor stimulus). All these quantities are unitless numbers since they are ratios from fluorescence intensities obtained from the microscope camera. We used the symbol [–] to represent this lack of units.

### Mathematical model

We used the SimBiology toolbox of Matlab (R2014a) to simulate the model. The details of the reactions implemented and rate constant used are provided in Additional file 1: Tables S1 and S2. To extract the MAPK activity based on the experimental nuclear-to-cytoplasmic ratio, an equation representing the MAPK activity as a function of time had to be provided. The parameters of this equation (Additional file 1: Table S3) were then optimized to obtain the best fit of the experimental data.

## Additional file

Additional file 1:
**Supplementary Text.** Description of the model. **Figure S1.** MAPK pathways in *S. cerevisiae.* **Figure S2.** Image segmentation process. **Figure S3.** Influence of the presence of SKARS on the pheromone response. **Figure S4.** Quantification of single cell responses. **Figure S5.** Slow responding cell in G2/M cell-cycle stage. **Figure S6.** Correlation between YFP and RFP sensors response. **Figure S7.** Optimization of Mpk1 sensor by adjusting the distance between docking site and the NLS. **Figure S8.** Single cell analysis of Mpk1 activation by zymolyase in low glucose concentration. **Table S1.** Reactions implemented in the model. **Table S2.** Kinetic parameters and starting concentrations. **Table S3.** Parameters describing MAPK activity dynamics. **Table S4.** List of yeast strains used in this study. **Table S5.** List of plasmids used in this study.

## Abbreviations

CWI: Cell wall integrity
DS: Docking site
FRET: Förster resonance energy transfer
HOG: High osmolarity glycerol
MAP2K: MAP kinase kinase
MAP3K: MAP kinase kinase kinase
MAPK: Mitogen activated protein kinase
ND: Non-docking (i.e. mutated Docking Site)
NLS: Nuclear localization sequence
SKARS: Synthetic kinase activity relocation sensor
WT: Wild-type

## References

[1] Qi M, Elion EA (2005). MAP kinase pathways. J Cell Sci.

[2] Sahoo R, Husain A, Elion EA (2009). MAP kinase in yeast.

[3] Bardwell L (2005). A walk-through of the yeast mating pheromone response pathway. Peptides.

[4] Pryciak PM, Huntress FA (1998). Membrane recruitment of the kinase cascade scaffold protein Ste5 by the Gbetagamma complex underlies activation of the yeast pheromone response pathway. Genes Dev.

[5] Levin DE (2005). Cell wall integrity signaling in Saccharomyces cerevisiae. Microbiol Mol Biol Rev.

[6] Bermejo C, Rodríguez E, García R, Rodríguez-Peña JM, Rodríguez de la Concepción ML, Rivas C (2008). The sequential activation of the yeast HOG and SLT2 pathways is required for cell survival to cell wall stress. Mol Biol Cell.

[7] Saito H (2010). Regulation of cross-talk in yeast MAPK signaling pathways. Curr Opin Microbiol.

[8] Patterson JC, Klimenko ES, Thorner J. Single-cell analysis reveals that insulation maintains signaling specificity between two yeast MAPK pathways with common components. Sci Signal. 2010;3:ra75. 10.1126/scisignal.2001275 PMC3995081 20959523

[9] Baltanás R, Bush A, Couto A, Durrieu L, Hohmann S, Colman-Lerner A. Pheromone-induced morphogenesis improves osmoadaptation capacity by activating the HOG MAPK Pathway. Sci Signal. 2013;6:ra26. 10.1126/scisignal.2003312 PMC3701258 23612707

[10] Ferrell JE, Machleder EM (1998). The biochemical basis of an all-or-none cell fate switch in Xenopus oocytes. Science.

[11] Paliwal S, Iglesias PA, Campbell K, Hilioti Z, Groisman A, Levchenko A (2007). MAPK-mediated bimodal gene expression and adaptive gradient sensing in yeast. Nature.

[12] Tomida T, Takekawa M, O’Grady P, Saito H (2009). Stimulus-specific distinctions in spatial and temporal dynamics of stress-activated protein kinase kinase kinases revealed by a fluorescence resonance energy transfer biosensor. Mol Cell Biol.

[13] Purvis JE, Karhohs KW, Mock C, Batchelor E, Loewer A, Lahav G (2012). p53 dynamics control cell fate. Science.

[14] Jans DA, Xiao CY, Lam MH (2000). Nuclear targeting signal recognition: a key control point in nuclear transport?. Bioessays.

[15] Harreman MT, Kline TM, Milford HG, Harben MB, Hodel AE, Corbett AH (2004). Regulation of nuclear import by phosphorylation adjacent to nuclear localization signals. J Biol Chem.

[16] Tanoue T, Nishida E (2003). Molecular recognitions in the MAP kinase cascades. Cell Signal.

[17] Enslen H, Davis RJ (2001). Regulation of MAP kinases by docking domains. Biol Cell.

[18] Tanoue T, Adachi M, Moriguchi T, Nishida E (2000). A conserved docking motif in MAP kinases common to substrates, activators and regulators. Nat Cell Biol.

[19] Yang SH, Galanis A, Sharrocks AD (1999). Targeting of p38 mitogen-activated protein kinases to MEF2 transcription factors. Mol Cell Biol.

[20] Reményi A, Good MC, Bhattacharyya RP, Lim WA (2005). The role of docking interactions in mediating signaling input, output, and discrimination in the yeast MAPK network. Mol Cell.

[21] Pelet S, Dechant R, Lee SS, van Drogen F, Peter M (2012). An integrated image analysis platform to quantify signal transduction in single cells. Integr Biol (Camb).

[22] Bishop AC, Ubersax JA, Petsch DT, Matheos DP, Gray NS, Blethrow J (2000). A chemical switch for inhibitor-sensitive alleles of any protein kinase. Nature.

[23] Pelet S, Rudolf F, Nadal-Ribelles M, de Nadal E, Posas F, Peter M (2011). Transient activation of the HOG MAPK pathway regulates bimodal gene expression. Science.

[24] English JG, Shellhammer JP, Malahe M, McCarter PC, Elston TC, Dohlman HG. MAPK feedback encodes a switch and timer for tunable stress adaptation in yeast. Sci Signal. 2015;8:ra5. 10.1126/scisignal.2005774 PMC4505820 25587192

[25] Macia J, Regot S, Peeters T, Conde N, Solé R, Posas F. Dynamic signaling in the Hog1 MAPK pathway relies on high basal signal transduction. Sci Signal. 2009;2:ra13. 10.1126/scisignal.2000056 19318625

[26] Oehlen LJ, Cross FR (1994). G1 cyclins CLN1 and CLN2 repress the mating factor response pathway at Start in the yeast cell cycle. Genes Dev.

[27] Wassmann K, Ammerer G (1997). Overexpression of the G1-cyclin gene CLN2 represses the mating pathway in Saccharomyces cerevisiae at the level of the MEKK Ste11. J Biol Chem.

[28] Strickfaden SC, Winters MJ, Ben-Ari G, Lamson RE, Tyers M, Pryciak PM (2007). A mechanism for cell-cycle regulation of MAP kinase signaling in a yeast differentiation pathway. Cell.

[29] Bean JM, Siggia ED, Cross FR (2006). Coherence and timing of cell cycle start examined at single-cell resolution. Mol Cell.

[30] Pramila T, Miles S, GuhaThakurta D, Jemiolo D, Breeden LL (2002). Conserved homeodomain proteins interact with MADS box protein Mcm1 to restrict ECB-dependent transcription to the M/G1 phase of the cell cycle. Genes Dev.

[31] Hao N, Yildirim N, Nagiec MJ, Parnell SC, Errede B, Dohlman HG (2012). Combined computational and experimental analysis reveals mitogen-activated protein kinase-mediated feedback phosphorylation as a mechanism for signaling specificity. Mol Biol Cell.

[32] Jiménez-Sánchez M, Cid VJ, Molina M (2007). Retrophosphorylation of Mkk1 and Mkk2 MAPKKs by the Slt2 MAPK in the yeast cell integrity pathway. J Biol Chem.

[33] Zarzov P, Mazzoni C, Mann C (1996). The SLT2(MPK1) MAP kinase is activated during periods of polarized cell growth in yeast. EMBO J.

[34] Kaffman A, O’Shea EK (1999). Regulation of nuclear localization: a key to a door. Annu Rev Cell Dev Biol.

[35] Fosbrink M, Aye-Han N-N, Cheong R, Levchenko A, Zhang J (2010). Visualization of JNK activity dynamics with a genetically encoded fluorescent biosensor. Proc Natl Acad Sci U S A.

[36] Ni Q, Titov DV, Zhang J (2006). Analyzing protein kinase dynamics in living cells with FRET reporters. Methods.

[37] Shaner NC, Steinbach PA, Tsien RY (2005). A guide to choosing fluorescent proteins. Nat Methods.

[38] Fritz RD, Letzelter M, Reimann A, Martin K, Fusco L, Ritsma L, et al. A versatile toolkit to produce sensitive FRET biosensors to visualize signaling in time and space. Sci Signal. 2013;6:rs12. 10.1126/scisignal.2004135 23882122

[39] Komatsu N, Aoki K, Yamada M, Yukinaga H, Fujita Y, Kamioka Y (2011). Development of an optimized backbone of FRET biosensors for kinases and GTPases. Mol Biol Cell.

[40] Görner W, Durchschlag E, Wolf J, Brown EL, Ammerer G, Ruis H (2002). Acute glucose starvation activates the nuclear localization signal of a stress-specific yeast transcription factor. EMBO J.

[41] Spencer SL, Cappell SD, Tsai F-C, Overton KW, Wang CL, Meyer T (2013). The proliferation-quiescence decision is controlled by a bifurcation in CDK2 activity at mitotic exit. Cell.

[42] Regot S, Hughey JJ, Bajar BT, Carrasco S, Covert MW (2014). High-sensitivity measurements of multiple kinase activitiesin live single cells. Cell.

[43] Sikorski RS, Hieter P (1989). A system of shuttle vectors and yeast host strains designed for efficient manipulation of DNA in Saccharomyces cerevisiae. Genetics.

[44] Longtine MS, McKenzie A, Demarini DJ, Shah NG, Wach A, Brachat A (1998). Additional modules for versatile and economical PCR-based gene deletion and modification in Saccharomyces cerevisiae. Yeast.

[45] Goldstein AL, McCusker JH (1999). Three new dominant drug resistance cassettes for gene disruption in Saccharomyces cerevisiae. Yeast.

[46] Sheff MA, Thorn KS (2004). Optimized cassettes for fluorescent protein tagging in Saccharomyces cerevisiae. Yeast.

[47] Edelstein A, Amodaj N, Hoover K, Vale R, Stuurman N. Computer control of microscopes using μManager. Curr Protoc Mol Biol. 2010;Chapter 14:Unit14.20. 10.1002/0471142727.mb1420s92 PMC3065365 20890901

