# Supplementary material for: Dynamic single cell measurements of kinase activity by synthetic kinase activity relocation sensors
Source: BMC Biol. 2015 Aug 1;13:55. doi: 10.1186/s12915-015-0163-z (PMC4521377; doi:10.1186/s12915-015-0163-z)
Supplement: Additional file 1: — Supplementary Text. Description of the model. Figure S1. MAPK pathways in S. cerevisiae. Figure S2. Image segmentation process. Figure S3. Influence of the presence of SKARS on the pheromone response. Figure S4. Quantification of single cell responses. Figure S5. Slow responding cell in G2/M cell-cycle stage. Figure S6. Correlation between YFP and RFP sensors response. Figure S7. Optimization of Mpk1 sensor by adjusting the distance between docking site and the NLS. Figure S8. Single cell analysis of Mpk1 activation by zymolyase in low glucose concentration. Table S1. Reactions implemented in the model. Table S2. Kinetic parameters and starting concentrations. Table S3. Parameters describing MAPK activity dynamics. Table S4. List of yeast strains used in this study. Table S5. List of plasmids used in this study. [file 12915_2015_163_MOESM1_ESM.pdf]

Dynamic single cell measurements of kinase activity by Synthetic Kinase Activity Relocation Sensors

Eric Durandau, Delphine Aymoz, Serge Pelet

Supplementary Materials

Supplementary Text: Description of the Model
Figure S1. MAPK pathways in *S. cerevisiae*
Figure S2. Image segmentation process
Figure S3. Influence of the presence of SKARS on the pheromone response
Figure S4. Quantification of single cell responses
Figure S5. Slow responding cell in G2/M cell-cycle stage
Figure S6. Correlation between YFP and RFP sensors response.
Figure S7 Optimization of Mpk1 sensor by adjusting the distance between docking site and the NLS
Figure S8. Single cell analysis of Mpk1 activation by zymolyase in low glucose concentration
Table S1: Reactions implemented in the model
Table S2: Kinetic parameters and starting concentrations
Table S3: Parameters describing MAPK activity dynamics.
Table S4. List of yeast strains used in this study
Table S5. List of plasmids used in this study

Supplementary Text: Description of the Model

Supplementary Table 1 lists the reactions implemented in the model. The sensor in the nucleus and in the cytoplasm is phosphorylated by the MAPK. We assume simple Michaelis-Menten kinetics for this reaction. The dephosphorylation is also modeled by a Michaelis-Menten law. We assume that these enzymes are processive and that the 4 phosphorylation events do not lead to ultra-sensitivity of the response.We think this is a fair assumption since during the development of the sensor we tested constructs with a variable number of phosphorylation sites and the general response of the sensor increased almost linearly with this number. We consider the amount of phosphatase and phosphatase activity to be constitutive. On the contrary, the MAPK activity changes with time. To account for this, V_{MAPK} is calculated as k_{Cat}*MAPK*$MAPK_{Activity}$. MAPK represents the total concentration of the kinase estimated to 0.7µM (Fus3+Kss1 ~14'000 molecules/cell, Volume of yeast cell ~35fl). The parameter $MAPK_{Activity}$ varies between 0 and 1 and represents the fraction of MAPK that are activated in the cell and thus can contribute to the phosphorylation of the sensor.

The sensor diffusion in and out of the nucleus has been estimated based on formula k_{Diff} = NPC/Na/Vn*p (Timney *et al. J Cell Biol* 2006, **175**:579–593). This determines the diffusion rate between nucleus and cytoplasm. Using the nuclear accumulation of the non-functional sensor, we can estimate that the active import of the sensor is 3.3 times larger than passive diffusion. The concentration of sensor was estimated to be relatively similar to the one of the MAPK (0.6µM). For starting condition, it is enriched almost three fold in the nucleus. Kinetic parameters and starting concentrations are listed in Table S2

For forward simulation of the model, the $MAPK_{Activity}$ evolves with time according to the formula:

$$MAPK_{Activity} = \frac{A}{1+e^{-R(t-T_0)}} + O$$

A: Amplitude of the response and varies from 0 to 1 in the different traces presented in Figure 3B

R: Rate of activation of the MAPK, set to 0.04

t: time axis

T_0: Start time, set to 950s

O: Offset value, set to 0

The model is simulated for 1800s. Time 900 is set to zero in the graphs from Figure 3B and C. The first 500s (-900 to -400) allow an equilibration of the SKARS between cytoplasm and nucleus.

To extract the $MAPK_{Activity}$ based on experimental data, Matlab optimizes the set of variables defining the variation of the $MAPK_{Activity}$ with time (*A*, *R*, T_0, *O*). For the dose response calculation (Figure 3E), we used the same equation as above. The nuclear to cytoplasmic ratio to be fitted consist in 5 time-points before zero set to the value of the active import and 8 time-points from 8 to 15 set to the nuclear to cytoplasmic ratio measured for one concentration of the dose response. The final value of the $MAPK_{Activity}$ after simulation is used as the steady-state MAPK ratio plotted in the dose response graph (Figure 3E).

To extract the $MAPK_{Activity}$ from the experimental data from Figure 2B a more complex equation was used to account for the rise and decay in $MAPK_{Activity}$:

$$MAPK_{Activity} = \frac{A_1}{1+e^{-R(t-T_0)}} + \frac{A_2}{1+e^{D(t-T_D)}} + O - 1$$

A_1 and A_2: Amplitude of the response

R and *D*: Rate of activation (deactivation) of the MAPK

t: time axis

T_0 and T_D: Start time and Decay time

O: Offset value

The time axis and median value of nuclear to cytoplasmic ratio is fed to the simulation. From an initial starting set of parameters, the program optimizes them to fit the experimental trace. The outcome of the fit is presented in Figure 3D. The starting and final parameters are given in Table S3.

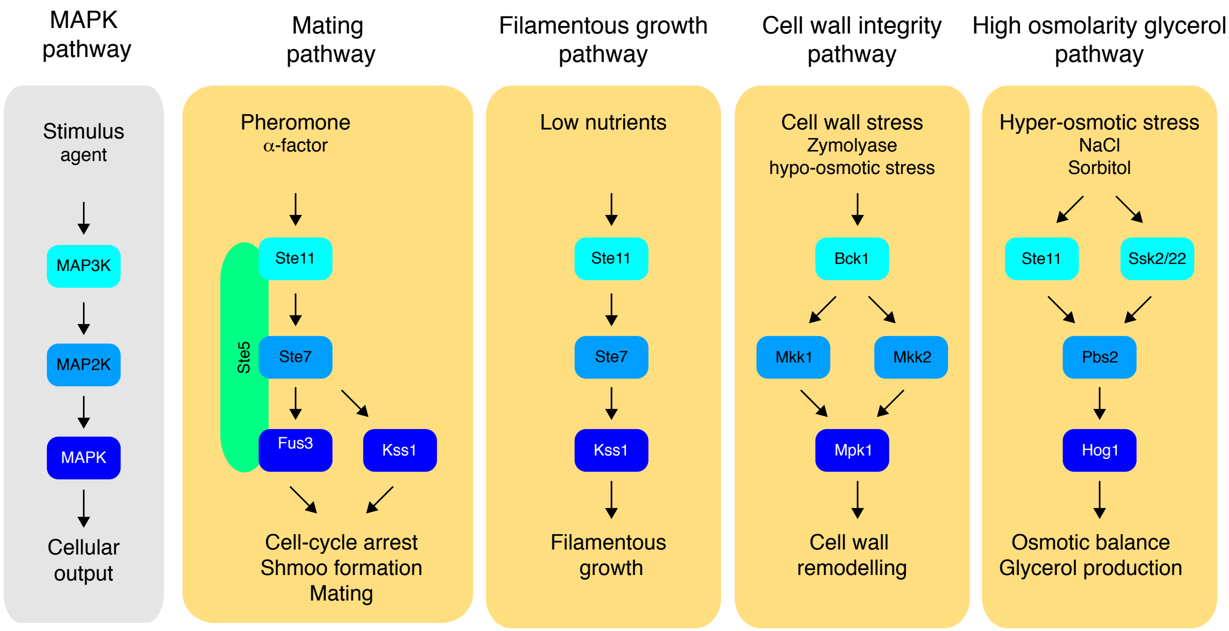

Figure S1. MAPK pathways in *S. cerevisiae*
General scheme depicting the four MAPK pathways active in haploid budding yeast cells: the mating pathway, the filamentous growth pathway, the Cell Wall Integrity (CWI) pathway and the High Osmolarity Glycerol (HOG) pathway.

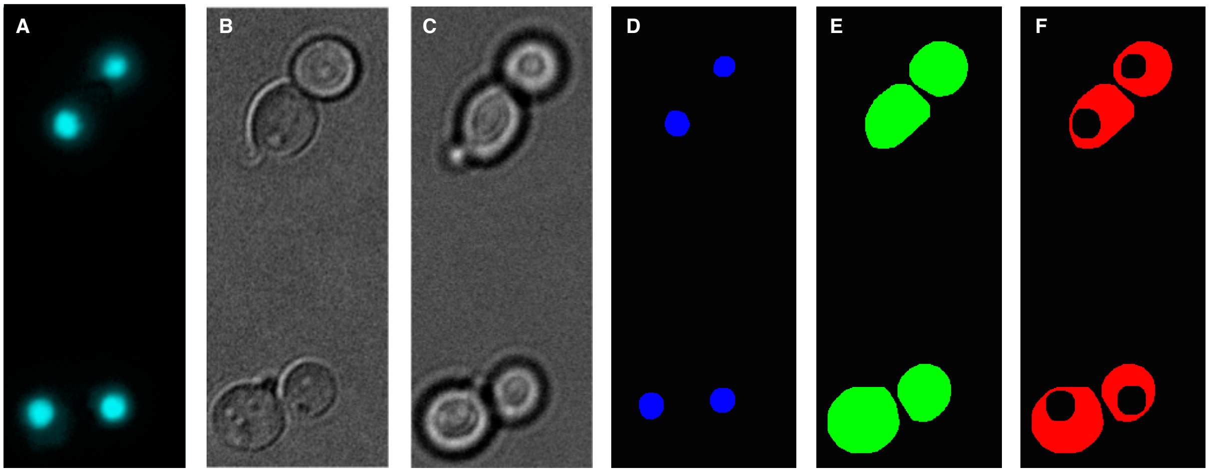

Figure S2. Image segmentation process
A, B and **C.** Three pictures are used for image segmentation: CFP: Hta2-CFP nuclear tag (A), BF0: in-focus brightfield image (B), BF1: slightly out of focus brightfield image (C). **D, E** and **F.** Based on these pictures, three objects are defined in the image. First, the Nucleus object is segmented based on the CFP intensity (D, blue). Then, using the two brightfield images, the contour of the cell is defined allowing to identify the Cell object (E, green). Finally, by subtracting the Nucleus object enlarged by 2 pixels from the Cell object, we define the Cytoplasm object (F, red).

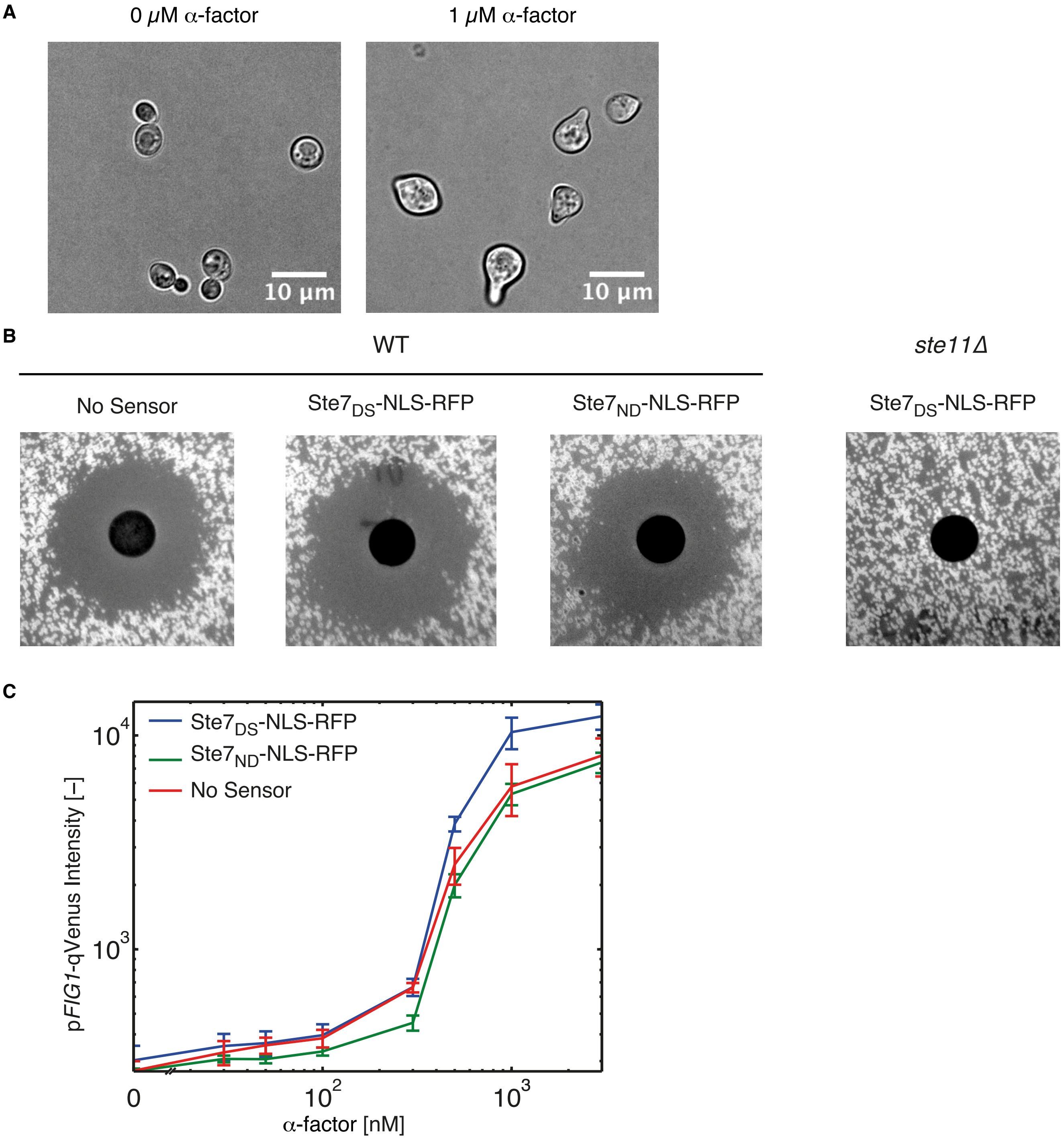

Figure S3. Influence of the presence of SKARS on the pheromone response.
A. Cells bearing the $Ste7_{DS}$-NLS-RFP sensor are able to form a mating projection upon α-factor stimulation. **B.** The ability of cell to arrest their cell cycle upon pheromone treatment was observed by halo assay. WT, $Ste7_{DS}$-NLS-RFP, $Ste7_{ND}$-NLS-RFP and $Ste7_{DS}$-NLS-RFP in *ste11*Δ cells were plated on rich medium. Cells were sowed with beads on a plate and 10μl of α-factor 1mg/ml was added on a filter disk. The absence of cell growth around the filter is indicative of the cell cycle arrest. The comparable size of the halo between cells bearing a functional sensor, a non-functional sensor or no sensor indicates an equivalent sensitivity to pheromone. **C.** Cells bearing an expression reporter p*FIG1*-qVenus where transformed with a functional ($Ste7_{DS}$-NLS-RFP) or non-functional ($Ste7_{ND}$-NLS-RFP) sensor. Cells were stimulated with a range of α-factor concentrations. 1.5 hours after stimulation they were treated with cycloheximide (0.1 mg/ml) and 1.5 hour later measured by flow cytometry. A minor increase in fluorescence level in cells bearing the functional sensor indicates a slight increase in expression ability of these cells.

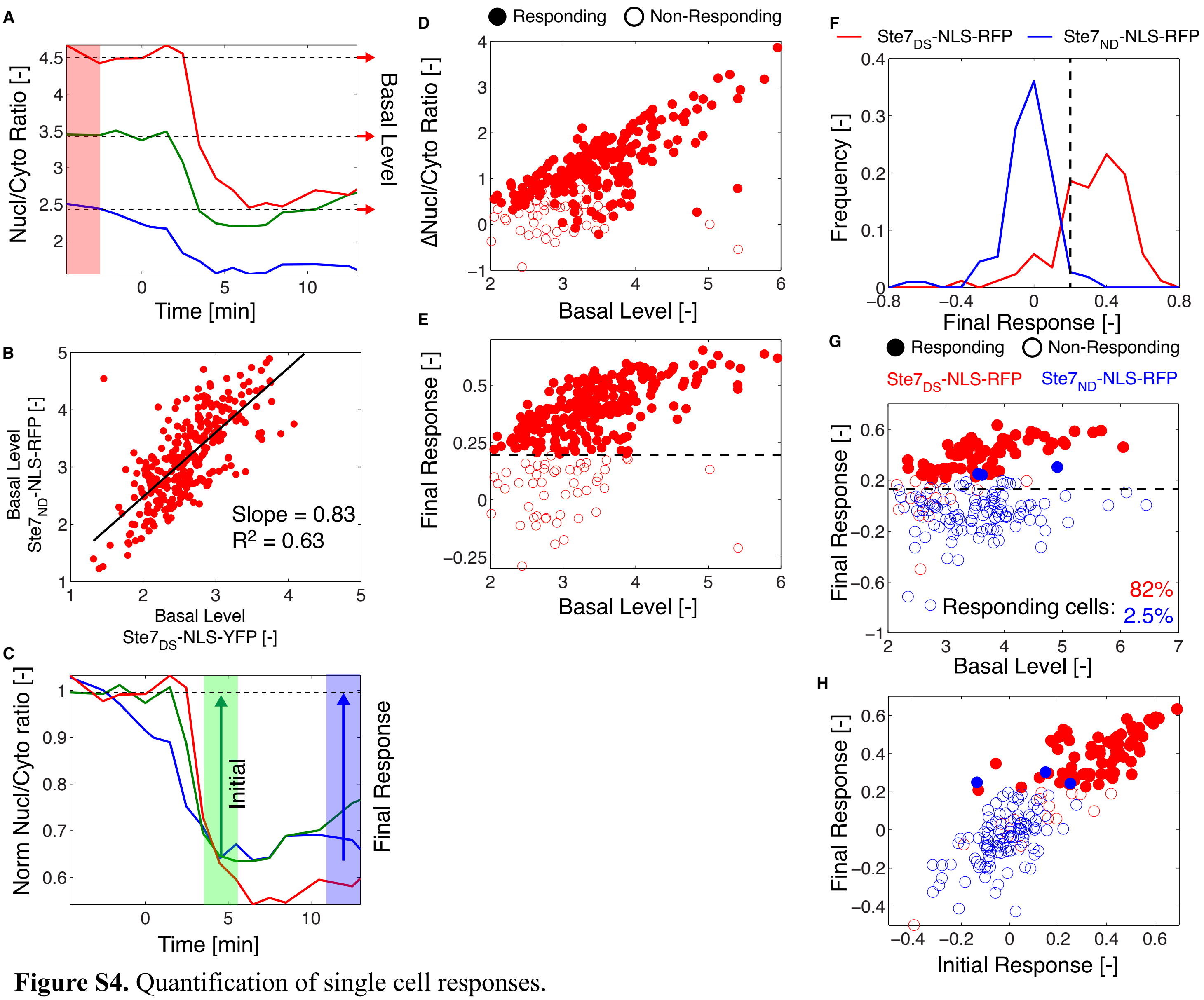

Figure S4. Quantification of single cell responses.

A. Nucleus to Cytoplasm ratio for three single cell traces. The mean value of the three first points allows to define the Basal Level of the trace (Nucl/Cyto$_{(t=-4:-2min)}$). **B.** Correlation between the Basal Level measured in the YFP and RFP channels of a strain bearing two sensors, one with a functional docking sequence (Ste7$_{DS}$-NLS-YFP) and one with a non-docking variant (Ste7$_{ND}$-NLS-RFP). The high degree of correlation between the two measurements demonstrates that the Basal Level of the sensor is mostly dependant on the intrinsic property of the cells to enrich the sensor and does not depend on a variable constitutive activity of the mating pathway. **C.** Single cell traces are normalized by their Basal Level to allow for a better comparison between individual cell responses. The normalized traces of the three same cells from panel A are shown in panel C. The Final Response is calculated as ([(Nucl/Cyto$_{(t=-4:-2min)}$) – (Nucl/Cyto$_{(t=12:15\ min)}$]/ [(Nucl/Cyto$_{(t=-4:-2min)}$)). The Initial Response is calculated as ([(Nucl/Cyto$_{(t=-4:-2min)}$) – (Nucl/Cyto$_{(t=4:6\ min)}$]/ [(Nucl/Cyto$_{(t=-4:-2min)}$)). **D** and **E.** Correlation between the Basal Level and the difference in Nucleus to Cytoplasm ratio [(Nucl/Cyto$_{(t=-4:-2min)}$) – (Nucl/Cyto$_{(t=12:15\ min)}$] (D) or Basal Level and the Final response (E). The strong bias for the difference in Nuclear/Cytoplasmic ratio as function of Basal Level is corrected by the normalization applied in panel E. Open circles are classified as non-responding cells. **F.** Histogram of the Final Response measured in a population of cells bearing the functional sensor (Ste7$_{DS}$-NLS-RFP, red) and the non-functional sensor (non-docking, Ste7$_{ND}$-NLS-RFP, blue). The histogram of the final response in the population allows to set a threshold (dashed line) at 0.2 to minimize the number of cells bearing the non-functional sensor considered as responding. **G** and **H.** Correlation between Basal Level and Final Response (G), and Final Response and Initial Response (H) for docking (red) and non-docking (blue) versions of the sensor. The filled circles represent the responding cells. 82% of the cells bearing the functional sensor are characterized as responding.These graphs demonstrate that the difference between a functional and non-functional sensor can also be observed at the single cell level.

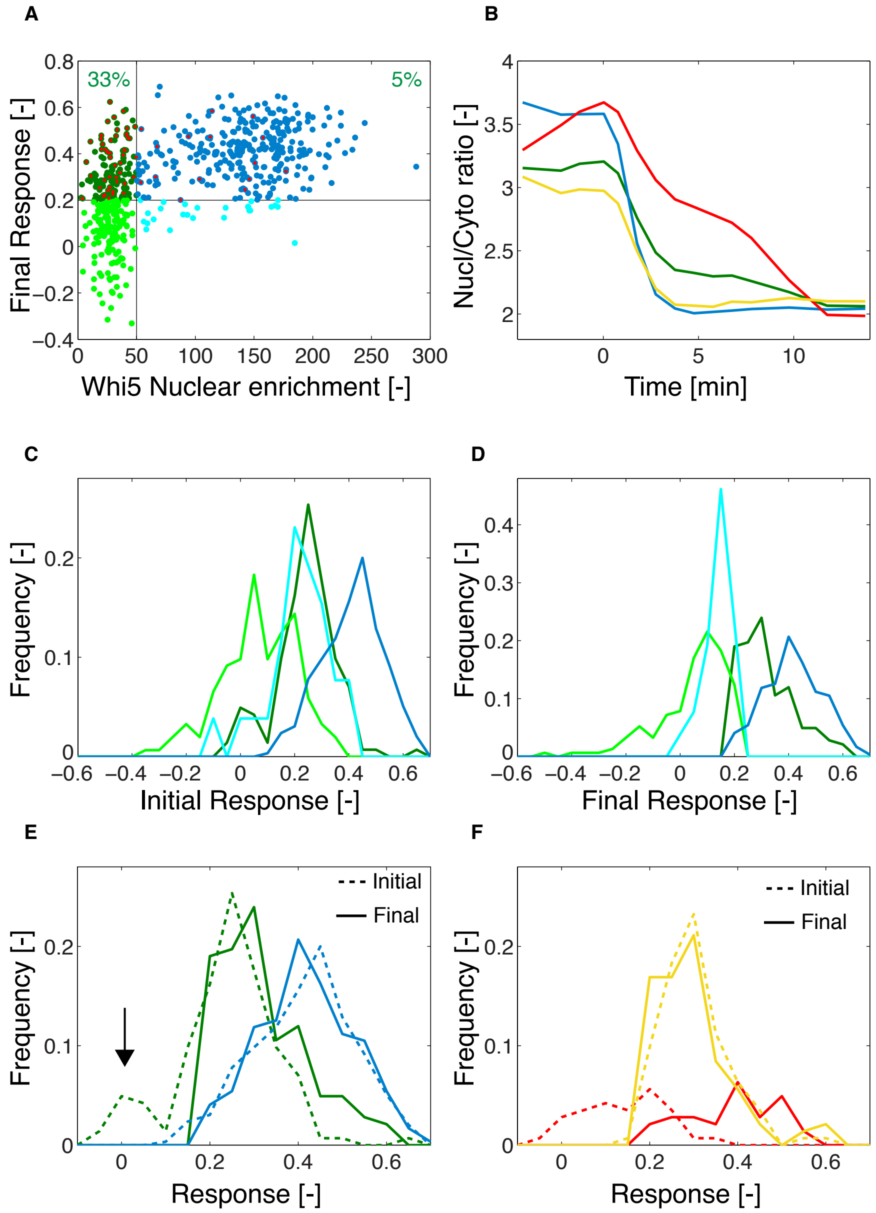

Figure S5. Slow responding cell in G2/M cell-cycle stage
A. Same panel as in Figure 5C displaying the Whi5 Nuclear enrichment vs. Final Response of the cell. Cells displaying a slow response are marked with a red dot. 33% of the G2/M cells are characterized as slow responding (only 5% in the G1 cells population). 76% of the slow responding cells are found in the G2/M quadrant. **B.** Dynamics of Nuclear exit of the SKARS. Blue: average response of G1 Cells. Green: average response of G2/M cells. Yellow: average response of the fast responding cells in G2/M stage. Red: average response of slow responding cells in G2/M stage. **C.** and **D.** Histograms of the Initial and Final Response of the four sub-population of cells selected in panel A. The histogram of the slow responding cells (dark gree) is the only one that shifts considerably between the initial and final response. **E.** Comparison of Initial and Final Response histograms for G2/M (green) and G1 (blue) cells. Note the shoulder in the histogram (arrow) denoting a population of cells that have not responded within the first 5 min of the time-lapse but which has diseappeared in the Final Response histogram. **F.** Comparison of Initial and Final Response histograms for G2/M cell with slow (red) or fast (yellow) response phenotypes. The slow responding cell display a clear shift in the histogram between Initial and Final Responses.

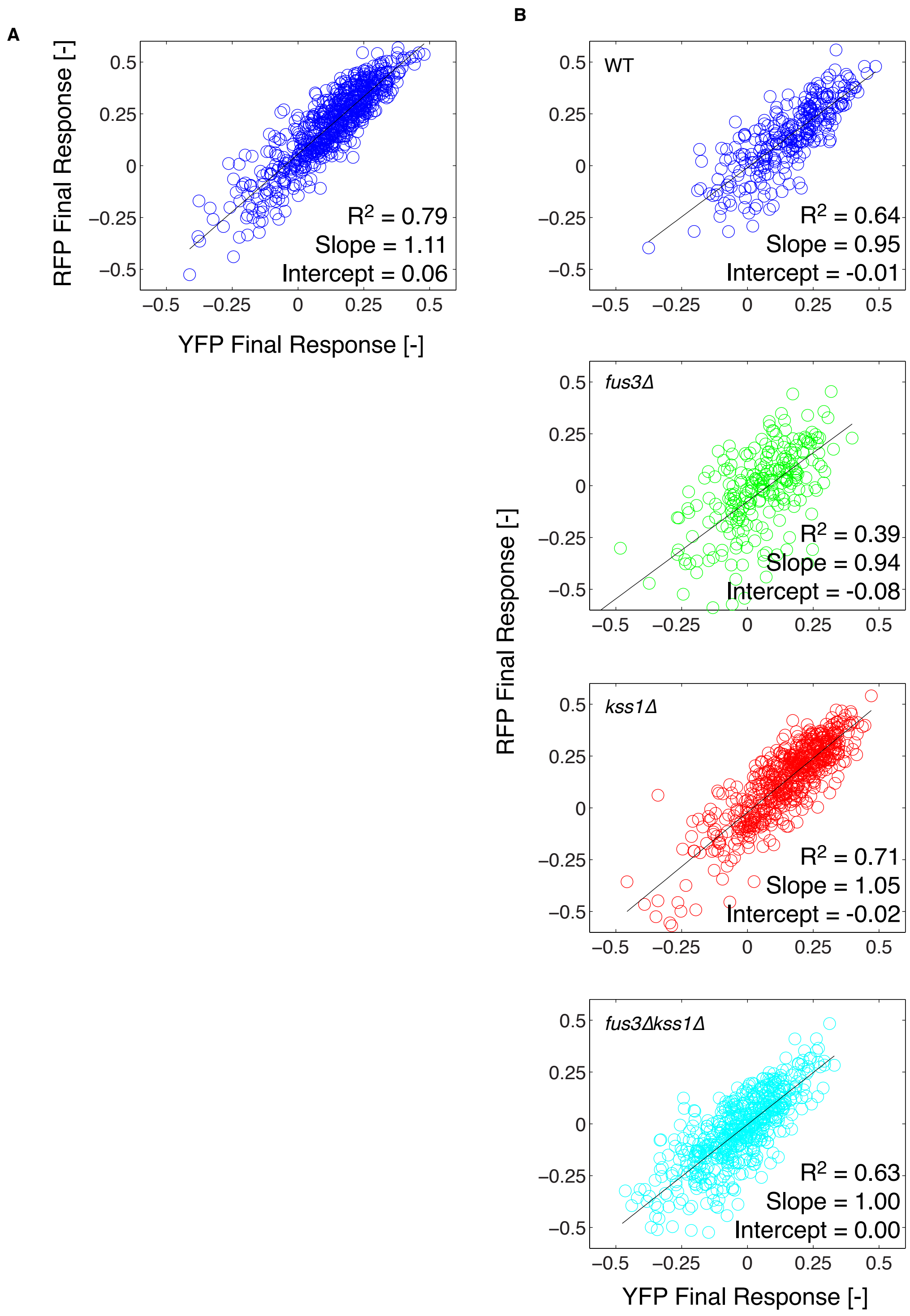

Figure S6. Correlation between YFP and RFP sensors response.
A. Correlation of the Final Response in cells bearing the $Ste7_{DS}$-NLS-YFP and $Ste7_{DS}$-NLS-RFP sensors. **B.** Correlation of the Final Response in cells bearing the $Ste7_{DS}$-NLS-YFP and $Far1_{DS}$-NLS-RFP sensors in WT and MAPK deletions. In the *fus3*Δ cells, the weakest correlation between the two measurements is observed.

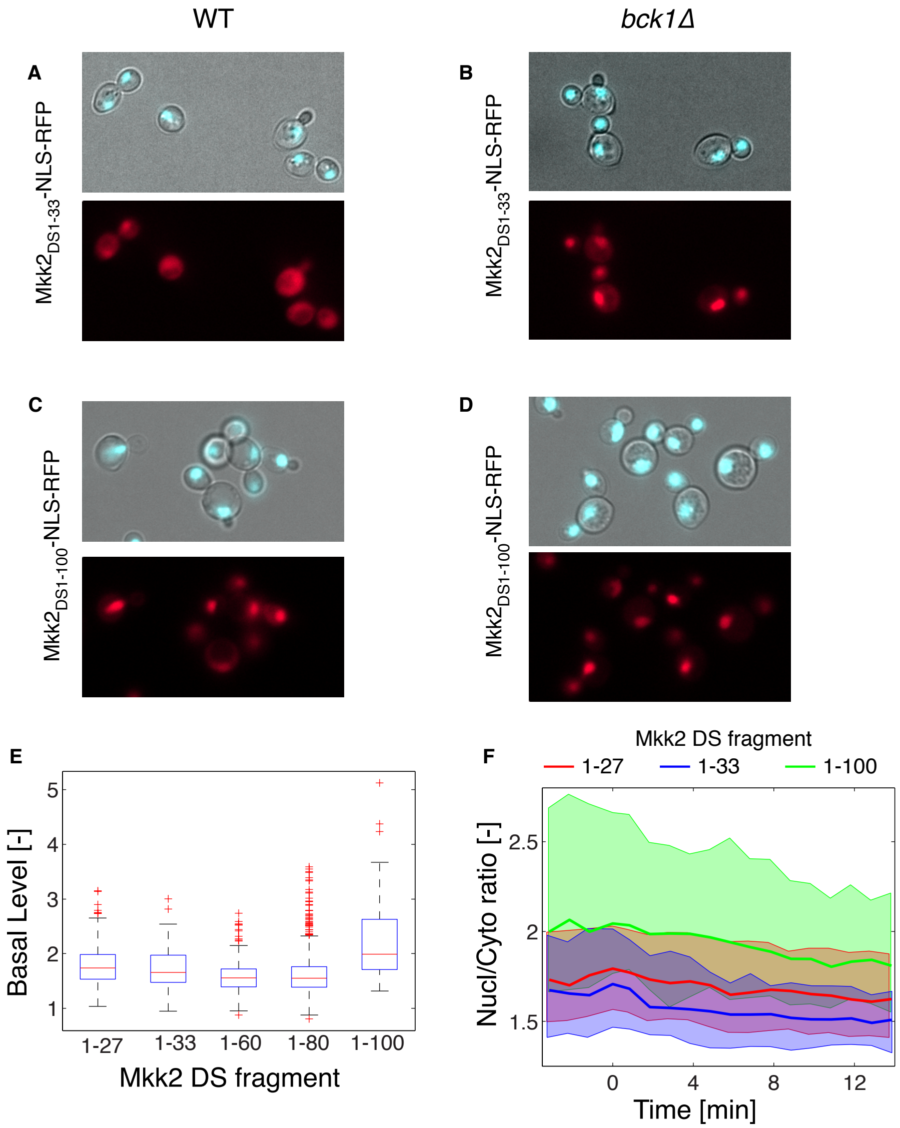

Figure S7. Optimization of Mpk1 sensor by adjusting the distance between docking site and the NLS. **A** and **B.** Images of WT (A) and *bck1*Δ (B) cells bearing the Mkk2$_{DS1\text{-}33}$-NLS-RFP sensor. The cytoplasmic localization of the sensor is signaling dependent. **C** and **D.** Images of WT (C) and *bck1*Δ (D) cells bearing the Mkk2$_{DS1\text{-}100}$-NLS-RFP sensor. The cells with the Mkk2$_{DS1\text{-}100}$-NLS-RFP sensor display a stronger basal nuclear enrichment than cells with shorter versions of the sensor. **E.** Quantification of the basal level of the sensor as function of the length of the Mkk2 fragment. **F.** Dynamics of response of three versions of the sensors upon zymolyase stress (3U/ml).

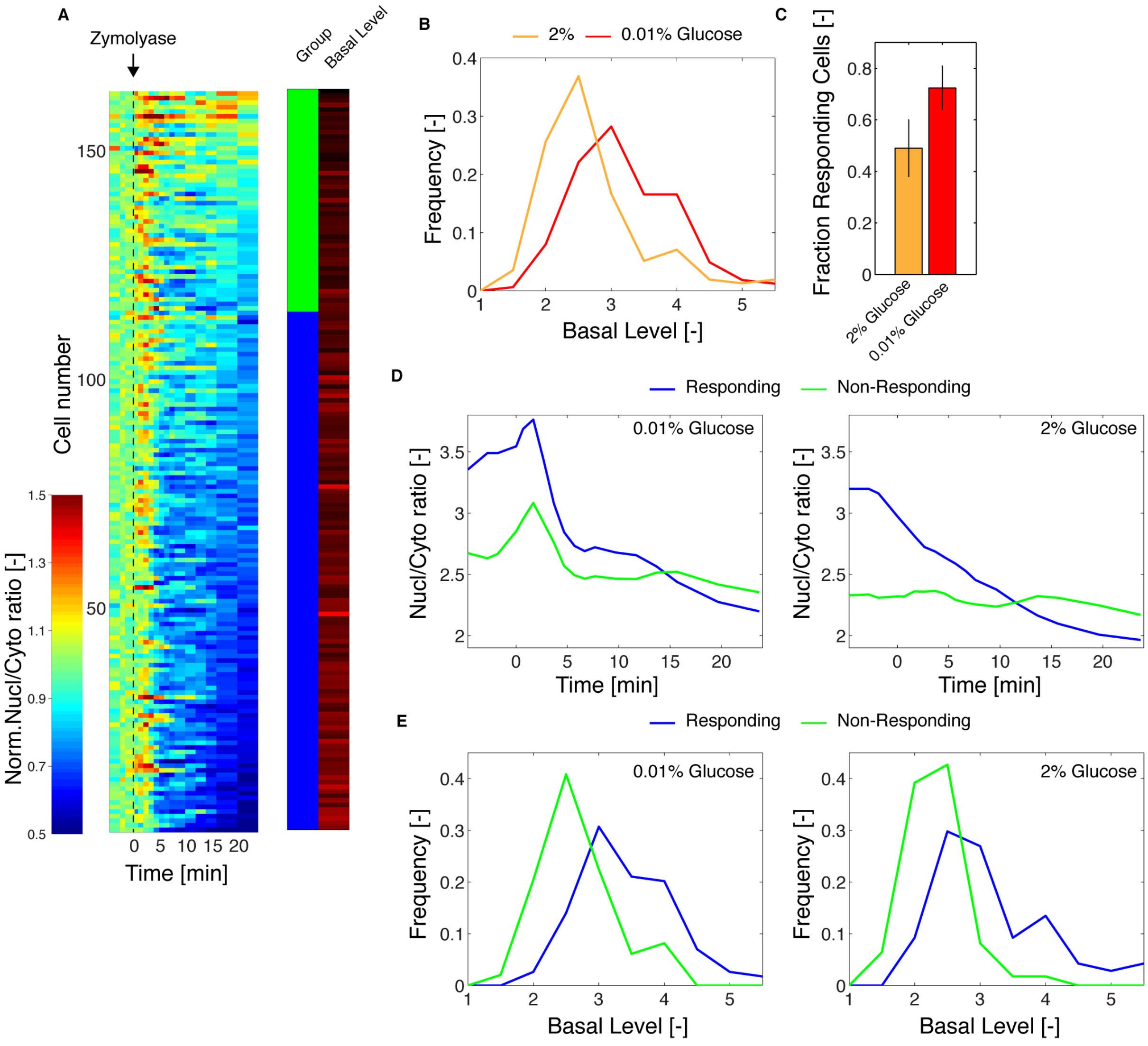

Figure S8. Single cell analysis of Mpk1 activation by zymolyase in low glucose concentration. **A.** Heat map of the response of individual cells bearing the SKARS Mkk2$_{DS1\text{-}100}$-NLS-RFP to zymolyase (3U/ml). Each line represents the normalized response of one single cell (N_c = 163). The cell traces were sorted based on the level of Final Response. This measurement was used to classify the cells in a group of responding cells (blue) and a group of non-responding cells (green). The Basal Level of each trace is indicated in shades of red (from black to red, increasing basal level). **B.** Histogram of the Basal Level of nuclear enrichement in 0.01% and 2% glucose. **C.** Fraction of responding cells in 2% and 0.01% glucose. Mean and standard deviation of four experiments are shown. **D.** Time-course of the Nuclear to cytoplasmic ratio for responding and non-responding cells at 0.01% and 2% glucose. **E.** Histogram of the Basal Level for responding and non-responding cells grown in 0.01% and 2% glucose.

Table S1: Reactions implemented in the model.

Reaction	Kinetic constant
$SKARS_{Cyto} \rightarrow SKARS_{Cyto}$-P	V_{MAPK}, K_{MAPK}
$SKARS_{Nucl} \rightarrow SKARS_{Nucl}$-P	V_{MAPK}, K_{MAPK}
$SKARS_{Cyto}$-P $\rightarrow SKARS_{Cyto}$	V_{PPase}, K_{PPase}
$SKARS_{Nucl}$-P $\rightarrow SKARS_{Nucl}$	V_{PPase}, K_{PPase}
$SKARS_{Cyto} \rightarrow SKARS_{Nucl}$	k_{Imp}
$SKARS_{Nucl} \rightarrow SKARS_{Cyto}$	k_{Diff}
$SKARS_{Cyto}$-P $\rightarrow SKARS_{Nucl}$-P	k_{Diff}
$SKARS_{Nucl}$-P $\rightarrow SKARS_{Cyto}$-P	k_{Diff}

Table S2: Kinetic parameters and starting concentrations

Parameter	Value	Unit
V_{MAPK}	k_{Cat} x MAPK x $MAPK_{Activity}$	µM/s
K_{MAPK}	0.5	µM
k_{Cat}	0.18	1/s
V_{PPase}	0.1	µM/s
K_{PPase}	0.5	µM
k_{Diff}	$NPC/N_A/V_N$*p ~ 0.05	1/s
NPC: number of nuclear pores	~ 160	
$N_{A:}$ Avogadro's Number	$6.022\ 10^{23}$	1/mol
V_N Volume of the Nucleus	3.5	fl
p: permissivity of nuclear pore	~ 0.06	1/µM/s
k_{Imp}	k_{Diff} x RatioActiveImport	1/s
RatioActiveImport	3.3	-
MAPK	0.7	µM
$MAPK_{Activity}$	0	-
$SKARS_{Nucl}$	0.43	µM
$SKARS_{Cyto}$	0.166	µM

Table S3: Parameters describing MAPK activity dynamics.

Parameter	Initial guess	Final value
A1	0.9	0.608
A2	1	0.772
R	0.01	0.041
D	0.2	0.0087
T_0	850	846
T_D	1500	1511
O	0.05	0.377

Table S4. List of yeast strains used in this study.

Strain	Background	Genotype	Plasmid	Figure
ySP2	W303	*MATa leu2-3,112 trp1-1 can1-100*<br>*ura3-1 ade2-1 his3-11,15*		
yED42	W303	*HTA2-CFP*<br>*ura3*:Ste7$_{DS}$-NLS-RFP	pED45	1B-F, 3D, 3E, 4A-F, S3A-B, S4A, S4C-H
yED54	W303	*HTA2-CFP*<br>*ura3*.Ste7$_{DS}$-NLS-RFP<br>*STE11::NAT*	pED45	1D, S3B
yED53	W303	*HTA2-CFP*<br>*ura3*:Ste7$_{ND}$-NLS-RFP	pED55	1E, 3E, S3B, S4F-H
yED51	W303	*HTA2-CFP*<br>*ura3*:Ste7$_{DS}$-NLS-4A-RFP	pED53	1F
yED52	W303	*HTA2-CFP*<br>*ura3*:Ste7DS-NLS-4E-RFP	pED54	1F
ySP456	W303	*HTA2-CFP*<br>*ura3*:Ste7$_{DS}$-NLS-RFP<br>*leu2:FUS3-as*<br>*KSS1::KAN FUS3::NAT*	pED45<br>pSP198	2A-B, 3F
ySP603	W303	*HTA2-CFP*<br>*ura3*:Ste7$_{DS}$-NLS-RFP<br>*WHI5*-mCitrine:*HIS3*	pED45	5A, 5C, 5E, S5A-F
ySP568	W303	*HTA2-CFP*<br>*ura3*:Ste7$_{DS}$-NLS-RFP<br>*trp1*:Ste7$_{DS}$-NLS-YFP	pSP303<br>pED73	6A, S6A,
ySP591	W303	*HTA2-CFP*<br>*ura3*:Ste7$_{DS}$-NLS-RFP<br>*YOX1*-mCitrine:*HIS3*	pED45	5B, 5D, 5F
ySP570	W303	*HTA2-CFP*<br>*ura3*:Far1$_{DS}$-NLS-RFP<br>*trp1*:Ste7$_{DS}$-YFP<br>*FUS3::NAT*	pSP303<br>pED73	6C-D, S6B

ySP571	W303	*HTA2-CFP*<br>*ura3*:Far1$_{DS}$-NLS-RFP<br>*trp1*:Ste7$_{DS}$-YFP<br>*KSS1::NAT*	 pSP303<br>pED73	6C-D, S6B
ySP572	W303	*HTA2-CFP*<br>*ura3*:Far1$_{DS}$-NLS-RFP<br>*trp1*:Ste7$_{DS}$-NLS-YFP<br>*KSS1::KAN FUS3::NAT*	 pSP303<br>pED73	6C-D, S6B
ySP569	W303	*HTA2-CFP*<br>*ura3*:Far1$_{DS}$-NLS-RFP<br>*trp1*:Ste7$_{DS}$-YFP	 pSP303<br>pED73	6C-D, S6B
ySP560	W303	*HTA2-CFP*<br>*ura3*:Mkk2$_{DS}$-NLS-RFP	 pSP323	7C-D, S7C, S7E-F, S8A-E
yED92	W303	*HTA2-CFP*<br>*ura3*:Mkk2$_{DS}$-NLS-RFP<br>*STE11::NAT*	 pSP323	7C-D
yED87	W303	*HTA2-CFP*<br>*ura3*:Mkk2$_{DS}$-NLS-RFP<br>*BCK1::NAT*	 pSP323	7C-D, S7D
ySP37	W303	*HTA2-CFP*		S3B
yED55	W303	*HTA2-CFP*<br>*ura3*:Ste7$_{DS}$-NLS-RFP<br>*leu2:pFIG1*-qVenus	 pED45<br>pSP31	S3C
yED56	W303	*HTA2-CFP*<br>*ura3*:Ste7$_{ND}$-NLS-RFP<br>*leu2:pFIG*1-qVenu*s*	 pED55<br>pSP31	S3C
yED57	W303	*HTA2-CFP*<br>*leu2:pFIG1*-qVenus	 pSP31	S3C
yED91	W303	*HTA2-CFP*<br>*ura3*:Ste7$_{ND}$-NLS-RFP<br>*trp1*:Ste7$_{DS}$-NLS-YFP	 pED55<br>pED73	S4B
ySP492	W303	*HTA2-CFP*<br>*ura3*:Mkk2$_{DS(1-33)}$-NLS-RFP	 pSP306	S7A, S7E-F
yED67	W303	*HTA2-CFP*<br>*ura3*:Mkk2$_{DS(1-33)}$-NLS-RFP<br>*BCK1::NAT*	 pSP306	S7B
ySP558	W303	*HTA2-CFP*<br>*ura3*:Mkk2$_{DS(1-60)}$-NLS-RFP	 pSP321	S7E-F
ySP559	W303	*HTA2-CFP*<br>*ura3*:Mkk2$_{DS(1-80)}$-NLS-RFP	 pSP322	S7E-F
ySP556	W303	*HTA2-CFP*<br>*ura3*:Mkk2$_{DS(1-27)}$-NLS-RFP	 pSP319	S7E-F
ySP596	W303	*HTA2-CFP*<br>*ura3*:Mkk2$_{DS}$-NLS-RFP<br>*WHI5*-mCitrine:*HIS3*	 pSP323	8A-D,
ySP594	W303	*HTA2-CFP*<br>*ura3*:Mkk2$_{DS}$-NLS-RFP<br>*YOX1*-mCitrine:*HIS3*	 pSP323	8E

Table S5. List of plasmids used in this study

Plasmid	Insert [a]	Backbone
pED45	p*RPS2*-Ste7$_{1\text{-}33}$-NLS-NLS-mCherry	pRS306
pED53	p*RPS2*-Ste7$_{1\text{-}33}$-NLS$_{(S14A,\ S22A)}$-NLS$_{(S14A,\ S22A)}$-mCherry	pRS306
pED54	p*RPS2*-Ste7$_{1\text{-}33}$-NLS$_{(S14E,\ S22E)}$-NLS$_{(S14E,\ S22E)}$-mCherry	pRS306
pED55	p*RPS2*-Ste7$_{ND}$-NLS-NLS-mCherry	pRS306
pED73	p*RPS2*-Ste7$_{1\text{-}33}$-NLS-NLS-mCitrine$_{A206K,\ L221K}$	pRS304
pSP31	p*FIG1*-quadruple Venus[c]	pRS305
pSP198	p*FUS3*-Fus3-as[b]	pRS406
pSP303	p*RPS2*-Far1$_{DS}$-NLS-NLS-mCherry	pRS306
pSP306	p*RPS2*-Mkk2$_{1\text{-}33}$-NLS-NLS-mCherry	pRS306
pSP319	p*RPS2*-Mkk2$_{1\text{-}27}$-NLS-NLS-mCherry	pRS306
pSP321	p*RPS2*-Mkk2$_{1\text{-}60}$-NLS-NLS-mCherry	pRS306
pSP322	p*RPS2*-Mkk2$_{1\text{-}80}$-NLS-NLS-mCherry	pRS306
pSP323	p*RPS2*-Mkk2$_{1\text{-}100}$-NLS-NLS-mCherry	pRS306

[a] NLS represents the peptidic sequence : `QQMGRGSEFEELGSPLKKLKISPDTASGLV`

Ste7$_{1\text{-}33}$ represents the peptidic sequence:

`MFQRKTLQRRNLKGLNLNLHPDVGNNGQLQEKT`

Ste7$_{ND}$ represent the peptidic sequence:

MFQRKTLQ**AA**NLKG**A**N**A**NLHPDVGNNGQLQEKT

Far1$_{DS}$ represents the peptidic sequence:

MFQRKTLQ**KRGNIPKPLNLSL**HPDVGNNGQLQEKT

[b] Macia *et al.*, *Science Signaling* **2:** ra13, 2009

[c] Pelet *et al.*, *Science* **332:** 732–735, 2011
